# Can an animal welfare risk assessment tool identify livestock at risk of poor welfare outcomes?

**DOI:** 10.1017/awf.2024.28

**Published:** 2024-09-16

**Authors:** Natarsha Williams, Sarah Chaplin, Lauren Hemsworth, Richard Shephard, Andrew Fisher

**Affiliations:** 1 Animal Welfare Science Centre, Faculty of Science, University of Melbourne, Parkville, VIC 3010, Australia; 2 Agriculture Victoria, Department of Energy, Environment and Climate Action, Tatura, VIC 3616, Australia; 3 School of Electrical and Data Engineering, Faculty of Engineering & IT, University of Technology Sydney, Sydney, NSW, Australia

**Keywords:** Animal welfare, extensive farming, livestock welfare, risk assessment, risk rating, welfare non-compliance

## Abstract

If livestock at risk of poor welfare could be identified using a risk assessment tool, more targeted response strategies could be developed by enforcement agencies to facilitate early intervention, prompt welfare improvement and a decrease in reoffending. This study aimed to test the ability of an Animal Welfare Risk Assessment Tool (AWRAT) to identify livestock at risk of poor welfare in extensive farming systems in Australia. Following farm visits for welfare- and non-welfare-related reasons, participants completed a single welfare rating (WR) and an assessment using the AWRAT for the farm just visited. A novel algorithm was developed to generate an AWRAT-Risk Rating (AWRAT-RR) based on the AWRAT assessment. Using linear regression, the relationship between the AWRAT-RR and the WR was tested. The AWRAT was good at identifying farms with poor livestock welfare based on this preliminary testing. As the AWRAT relies upon observation, the intra- and inter-observer agreement were compared in an observation study. This included rating a set of photographs of farm features, on two occasions. Intra-observer reliability was good, with 83% of Intra-class Correlation Coefficients (ICCs) for observers ≥ 0.8. Inter-observer reliability was moderate with an ICC of 0.67. The AWRAT provides a structured framework to improve consistency in livestock welfare assessments. Further research is necessary to determine the AWRAT’s ability to identify livestock at risk of poor welfare by studying animal welfare incidents and reoffending over time.

## Introduction

Incidents of poor livestock welfare on farms in Australia are typically investigated by authorised officers under the relevant animal welfare legislation (Department of Energy, Environment and Climate Action [DEECA] 2023). In Australia, the legislation varies with each state and territory (Department of Agriculture, Water and the Environment [DAWE] 2019). An animal welfare investigation typically involves a visit to the farm and an inspection of the animals to determine if there has been a breach in the legislation (DEECA 2023). Examples of non-compliance include a failure to provide proper and sufficient food, treatment or veterinary attention (Victoria State Government 1986). Officers then work with the farmer to resolve the issues by providing advice on the measures required to improve the health and welfare of the animals (Royal Society for the Prevention of Cruelty to Animals [RSPCA] 2022; DEECA 2023). Once there is no longer a breach of the legislation, the on-farm investigation is complete. Regulatory outcomes may be applied, such as advisory letters, warning letters and infringements. In serious incidents, cases may be prosecuted (Morton *et al.* 2020; RSPCA 2022; DEECA 2023), and sometimes the livestock will be seized (RSPCA 2022; DEECA 2023). Instances of livestock welfare non-compliance on-farm mostly result from neglect rather than a malicious act (Hedman *et al.* 2018; Morton *et al.* 2018; Sentencing Advisory Council [SAC] 2019; Temple & Manteca 2020; Väärikkälä *et al.* 2020; Williams *et al.* 2024b) and 27% of people identified with poor livestock welfare in one study reoffended (Williams *et al.* 2024b). Currently, to the authors’ knowledge, there is no systematic mechanism for assessing welfare risk during farm visits. Investigations typically focus upon evidence that relates to the relevant breach(es) of the animal welfare legislation – for example, animals in paddocks with no pasture or supplementary feeding or injured animals that have not been treated (Williams *et al.* 2024b).

An Animal Welfare Risk Assessment Tool (AWRAT) was developed by the authors in a previous study published in conjunction with this one (Williams *et al.* 2024a). It includes risk factors commonly observed on properties where the welfare of the livestock is poor (Williams 2024). It is proposed that by considering the circumstances in which the livestock live, the ongoing risk of poor welfare may be determined. Some of the risk factors, for example, a lack of infrastructure and basic management such as controlled breeding and weaning, can make it more challenging to provide the appropriate care of livestock. Other risk factors are also a direct cause of the problem (Schooling & Jones 2018), for example, lack of nutrition. To identify the risk of livestock having poor welfare, a tool would require the flexibility to be able to create a risk rating even when not all the factors in the tool have been assessed. This is important because not all risk factors would be observed on every farm visit.

Having a systematic framework in which to perform livestock welfare assessments would provide a more standardised and objective approach to welfare assessment, enabling greater consistency when successive farm visits are undertaken by different staff. Such a tool would also assist in broadening the observational skills of inspectors, and prompt consideration of less obvious factors that might contribute to poor management and welfare outcomes. Less experienced staff may also benefit from having a defined structure of assessment and it would assist in calibrating assessments between inspectors. If it were possible to identify situations where the risk of poor welfare was high on the first inspection or during routine non-welfare-related visits, instances of poor welfare and reoffending may be prevented by targeted intervention (The Farm Animal Welfare Advisory Council [FAWAC] 2018). Additionally, a measure of risk could be used to manage response priorities (Sandgren *et al.* 2009), frequency of revisits and the use of legal instruments. The risk assessment could also facilitate a discussion with the farmer about managing and reducing the risk to animal welfare (Zsidisin *et al.* 2004). In cases prosecuted in court, the assessment may be used to inform sentencing (Williams *et al.* 2024a), as has been the case in other disciplines (Kleiman *et al.* 2007; van Ginneken 2019). Lastly, repeating risk assessments in investigations that are prolonged may provide a way to monitor change and improvement and focus response efforts in difficult cases (Williams *et al.* 2024a).

Risk assessment can be used to predict and/or identify a number of outcomes (Kleiman *et al.* 2007; Desmarais *et al.* 2012; Garrett & Monahan 2019), by measuring the likelihood, without certainty, of a defined problem occurring (Bureau of Justice Assistance [BJA] 2023). Assessments include risk factors that are known to be correlated with the problem outcome (O’Connell *et al.* 2009; Garrett & Monahan 2019). It has been proposed that there are factors that are more commonly observed on farms where the welfare of the livestock is poor, and these may be considered risk factors (Williams *et al.* 2024a).

The AWRAT relies upon assessors observing and rating the relevance of a number of factors about the farm, nutrition and the animals on a visual analogue scale (Williams *et al.* 2024a). Observation is the key skill required for the AWRAT and it needs to be reliable and repeatable within and between individuals. As part of the development of any new measurement tool, an observer variability assessment (Popović & Thomas 2017) should be completed. Intra-observer reliability tests assess the similarity in results by the same observer in the same situation at different times. Secondly, inter-observer reliability tests the similarity of two different observers in the same situations at the same time (Pfeifer *et al.* 2019; Harrell 2022).

This study aimed to examine the AWRAT’s ability to identify livestock at risk of poor welfare. Ideally, this would have included a comparison with a gold standard test. Without such a test available the study compared an overall, subjective animal welfare rating (WR) provided by each study participant prior to completing the detailed, systematic AWRAT assessment. As the AWRAT is based on observation, this study also aimed to compare the intra- and inter-observer agreement based on an observation study. It is proposed that by observing and rating factors about the farm, nutrition, management/husbandry, treatment and the animals themselves, it may be possible to identify livestock at risk of poor welfare.

## Materials and methods

### Ethical approval

Human research ethics approval was obtained from the University of Melbourne’s Human Ethics Advisory Group (Ethics ID: 24351) and informed consent was obtained from all subjects involved in the study.

### Methodology

This AWRAT study included two components: Animal Welfare Risk Assessment Tool trial (AWRAT trial) and the Observation Study (OS). The AWRAT trial was designed to test the effectiveness of the AWRAT at identifying livestock at risk of poor welfare and the OS was designed to compare the intra- and inter-observer agreement of observation based on photographs of different scenarios.

### Recruitment

Agencies that have a responsibility to perform livestock animal welfare investigations and animal health or extension visits to farms from all Australian jurisdictions were invited to participate in both parts of the AWRAT study. Relationships with many of these groups had already been established through a previous survey conducted by the authors (Williams *et al.* 2024a). Contact details for invitees were identified through known contacts, public records and sharing of information between similar groups. Initial contact and reminders were sent via email. To be eligible, participants needed to be at least 18 years old, willing to be involved, with a minimum of six months’ experience working with non-dairy cattle, sheep and goats and hold a work role that included farm visits. Participants were also required to have access to a smart phone or computer and have suitable basic skills in their use. Involvement in the AWRAT study was voluntary and anonymous.

Participants were briefed on the study by telephone, video meeting or email. Briefings included background on the study, what was being asked of them, the safety instructions, as well as an opportunity to ask questions.

### Study design

After the briefing participants were sent access to and instruction for the AWRAT trial and OS. Both sections of the study were accessed separately on the Qualtrics survey platform (Qualtrics, Provo, UT, USA) via a link and QR code using a smart phone, tablet or computer. All survey sections were tested by three people that were suitably qualified prior to the onset of the study.

#### The AWRAT trial

The AWRAT was developed in a previous study by the authors (Williams *et al.* 2024a) and a copy of the tool can be found in the Supplementary materials. Participants were asked to complete a farm assessment using the AWRAT after every farm visit they completed over the study period. This applied to welfare- and non-welfare-related visits and revisits to farms where there were at least ten extensively managed non-dairy cattle, sheep or goats. The first part of the AWRAT included five questions which were used to create an anonymous code for the participant and the farm. There were also questions regarding the reason for the visit and any history of prior offences. Participants were also asked: ‘*Using the scale below mark the spot that you feel best describes the overall welfare status of the livestock on the farm during your visit today?*’ The scale was marked with very poor on the left, corresponding to 0 and very good on the right, corresponding to 100. The position marked on the scale by the participants was automatically converted into a number between 0–100 and this was the welfare rating (WR). This WR was used to compare to the AWRAT assessment. In the AWRAT trial, ratings were provided on a visual analogue scale with no markers except ‘not true’ on the left, corresponding to 0 and ‘very true’ on the right, corresponding to 100. Participants were provided with a detailed standard operating procedure (SOP) explaining the use of the AWRAT and were asked to read it before completing any assessments. The SOP included details of possible observations that might assist in validating the relevance of each factor on-farm. The SOP is included in the Supplementary material.

To ensure that there were no additional safety risks, participants were asked not to consult with the farmer or to travel to parts of the farm not otherwise required, when completing the assessment. Farmers are not always present during visits, but interactions with them can be hostile when the nature of the visit is compliance. Participants were also encouraged not to guess the relevance of any of the factors and were advised that it was not necessary to provide a rating for all factors in the assessment for a risk assessment to be generated. This was because it would not always be possible to rate all of the factors on genuine farm visits. The flexibility of the AWRAT to measure risk when not every risk factor is considered is a key feature of the tool and examining this flexibility was an important aspect of the trial.

The AWRAT study ran from 24/8/22–30/4/23, but recruitment was staggered over this period, as it took longer to engage some agencies more than others.

#### Observation study (OS)

The OS compared the intra- and inter-observer agreement via an online test using photographs of various farm features. The majority of the images were accessed through Wikimedia commons (Foundation 2023) and were used under a Creative Commons Licence (Commons 2023) others were provided by an anonymous source. Each photograph had an accompanying statement, which referred to the suitability of the feature in the image to a particular livestock farming enterprise. For example, an image of fencing on a farm and a comment ‘*For a sheep enterprise there is evidence that the farm fencing is of an effective standard (e.g. stock proof, swinging gates)’.* Participants were asked to rate how accurately they thought the statement reflected what they saw in the image using a visual analogue slider scale. The slider had no scale, but the extremes were marked as ‘not accurate’, corresponding to 0, and ‘accurate’, corresponding to 100 and the pointer was placed in the middle. The rating selected by the participants was automatically converted to a number from 0–100 by the survey platform. To examine intra-observer reliability (i.e. the stability of responses obtained from the same respondent at different time-points) (Bateson & Martin 2021), participants were asked to complete the OS test twice, no less than ten days apart. Ten days were chosen between tests to reduce the likelihood that participants would remember the exact ratings they used in their first test while also keeping them engaged with the study. In the first section of the OS, participants were asked three questions, the answers of which created an anonymous code, allowing the researcher to identify tests completed by the same participant, without being able to personally identify them. In addition, participants’ main work role and location were recorded. A full copy of the OS test can be seen in the Supplementary material.

### Analysis

#### The AWRAT trial

The assessments from the AWRAT study were downloaded from Qualtrics in a Microsoft Excel® spreadsheet. Entries with no values, no WR or relating to non-key livestock were removed. Questions about the participants’ location, work role, reason for the farm visit and the WR were collected in the first section. In the second section (the AWRAT assessment), the data were divided into two sets. The trial data-set (n = 71) were collected from 24/8/22 to 12/1/23 and used to derive a suitable algorithm to measure risk. Secondly, the test data-set (n = 40) were collected while the algorithm was being developed, between 13/1/23 to 30/4/23 and used to test the algorithm. The duration of the collection period was set to ensure there were enough data in both sections for analysis. Ninety-two percent of entries in the trial data-set were suitable for analysis and 67% in the test data-set. Discussion on the different seasons in which this data were collected occurs later.

In the test data-set there were a number of AWRAT assessments that had been entered retrospectively for farm visits that had occurred up to three months previously. Only assessments that were recorded within one month of the visit were analysed, and only one assessment per property if multiple previous revisit assessments were recorded on the same day. This was done to ensure that the analysis did not compare assessments made too long after the farm visits and to avoid analysis of duplicated assessments. Officers have investigation records that they could have drawn upon to assist with completing assessments for visits that had occurred up to one month previously.

### Development of AWRAT algorithm

The development of an algorithm to derive a measure of risk from the AWRAT assessments involved a number of steps and trial and error.

### Assigning a value to the factors

The 18 risk factors from the AWRAT were regrouped into four topic areas: farm, animals, treatment and nutrition, which made it easier to consider the likely welfare impact of each group of risk factors. Each topic area was assigned a topic-risk rating (TRR) using a subjective estimation of the overall impact and importance of each factor in the topic area on livestock welfare. The TRR was developed as a weighting method to adjust the algorithm (Mercer *et al.* 2018) to allow for all factors in the AWRAT not posing an equal risk on livestock welfare. The TRR was a proportion of 100, where the sum of the TRR for each topic area was 100. The TRR was determined subjectively by considering which factors were more commonly associated with the most severe welfare cases and reoffending in a future study (Williams *et al.* 2024b). Additionally, the direct and indirect impact of the factors on livestock welfare, the irreversibility of the issue including the time, cost and effort to improve the issue and the chronicity of the problem were considered. As there were four topic areas, each would have a TRR of 25 if they had an equal impact on the welfare outcomes. Failing to provide adequate treatment was commonly identified in the least and most severe welfare cases, the issues were relatively easy, inexpensive and quick to resolve, the topic area ‘treatment’ was given a TRR of 20. Problems with nutrition were significantly more common in more severe welfare cases and reoffending but are relatively uncomplicated and fast to resolve. Increasing feed can be expensive but only a small proportion of the cost required to improve farming infrastructure in comparison. Nutrition issues can also be alleviated by decreasing the number of animals, and selling animals generates income. The TRR for nutrition was 20. The farm and animal factor topic areas were assigned a TRR of 30 each. The farm factors were mostly associated with a lack of maintenance and upkeep on the farm, and it is likely this has occurred over an extended period. Stock fences and stock handling infrastructure are expensive and time consuming to repair. Without suitable infrastructure, management/husbandry procedures are difficult to conduct, therefore the impact upon livestock welfare can be significant and ongoing. The animal factors largely resulted from a lack of basic husbandry and management. Although the problems themselves are relatively easy to fix, by selling stock, and managing males better, the impact upon welfare can be severe and ongoing as animals take a long time to recover. Failing to perform routine procedures may also indicate a sustained failure to provide care. Measures such as unsuitable use of males, overstocking, failing to wean, dip/drench and draft were all significantly more common in severe welfare cases and reoffending.

### Measure of completeness (MOC)

Participants were advised they did not need to provide a rating for every factor in the AWRAT, resulting in a large number of missing values. The Measure of Completeness (MOC) was developed to assess the impact of the missing values on the reliability of the risk assessment. Each factor was assigned a factor value, which was the TRR for the topic area, divided by the number of factors in that topic area (see Table 1). The MOC was a weighted number, as the number of factors in each topic area were different, and the impact on welfare factors was not the same, as explained above. The maximum MOC was 100, when all factors had a rating and the lowest possible was zero, where there were no ratings.

**Table 1.** AWRAT factors, Topic Risk Rating (TRR) and factor values for each topic area

AWRAT factors, by topic area	TRR	Factor value (each)
Farm		
1.1 – There is evidence that the farm fencing is of an effective standard (e.g. stock proof, swinging gates) 1.2 – There is evidence that the stock handling facilities are suitable and in working order 1.3 – There is evidence that the feed and water areas and facilities are clean, functional and accessible to all livestock 1.5 – There is evidence that paddocks with livestock are suitable to avoid illness and accidental injury (e.g. toxic plants, feeding off the ground)	30	7.5
Treatment		
3.2 – There is evidence that animals with conditions that are unresponsive or not economical to treat are humanely culled without delay (e.g. lameness, cancers, chronic scouring, congenital abnormalities) 3.3 – There is evidence that the investigation and treatment of health conditions affecting livestock are completed as soon as the problem is identified	20	10
Animals		
1.6 – There is evidence that dead animals are removed from paddocks 3.1 – There is evidence that the lambs, calves and/or kids within a mob/herd are all a similar age (within 2–3 months of each other) 3.4 – There is evidence that entire male livestock are securely managed away from female stock that are too young and /or too small to be pregnant 3.6 – There is evidence that the farm is stocked with the appropriate number of livestock for the area available (not overstocked)	30	7.5
Nutrition		
1.4 – There is evidence that the farm has an equivalent amount of pasture (or better) to that on surrounding farms 2.1 – There is evidence that livestock are fed according to their reproductive state (e.g. empty, pregnant or lactating) 2.2 – There is evidence that some paddocks have good feed remaining 2.3 – There is evidence that the feed offered is suitable for the class of animal (e.g. energy, protein etc) 2.4 – There is evidence that supplementary feeding is offered before significant weight loss occurs 2.5 – There is evidence that there is some supplementary feed remaining in the paddock after the livestock have finished feeding 2.6 – There is evidence that all animals have equal access to supplementary feeding (e.g. well spread out or continuous access) 3.5 – There is evidence that livestock in poorer condition are drafted out of the mob/herd/ flock and preferentially fed	20	2.5
Total	100	100

### Calculation of an AWRAT-Risk Rating (AWRAT-RR) and MOC

The final step in the analysis of the AWRAT trial was to develop an AWRAT-risk rating (AWRAT-RR); a number from 0–100 that is a measure of risk of livestock having poor welfare, where 0 is an extremely high risk of poor welfare and 100 is an extremely low risk of poor welfare. The algorithm to turn the AWRAT assessment into a risk rating was written in Microsoft Excel®. In order for ratings of zero to be considered as a value, all zeros were changed to 0.01. For each topic area, only the factors that had ratings were considered initially: all the ratings were added together and divided by the maximum possible rating (for those with ratings) for each topic area. The resulting fraction was multiplied by the TRR for that topic area (as listed in Table 1). The resulting number for all topic areas were added together to generate the AWRAT-RR. To calculate the MOC, the number of factors without ratings in each topic area was multiplied by the factor value. This value was subtracted from the TRR for each topic area, and the resulting values for all topic areas were added together, to calculate the MOC.

All the entries in the trial data-set were analysed using the AWRAT and MOC algorithm to create an AWRAT-RR and MOC. Linear regression analysis was used to determine the relationship between the AWRAT-RR and the WR provided by the officers at the time of the visit. Firstly, a relationship was tested for all assessments in the trial data-set, regardless of the MOC. Secondly, separate regression analysis was completed for assessments for which the MOC was ≥ 70, ≥ 80, ≥ 90 and 100. These MOCs represented assessments where the majority (MOC = 70, 80, 90) or all (MOC = 100) the questions had been answered. This process was then completed using the test data-set.

#### The observation study

The responses were downloaded from Qualtrics into a Microsoft Excel® spreadsheet. The total number of observers that had completed the OS once or twice were calculated. An Intra-class Correlation Coefficient (ICC) was used to determine the intra- and inter-observer reliability. This method was selected as the observations were ratings from 0–100 and the authors wanted to maintain that detail in assessing observer reliability. ICC has also been recommended for this type of data in the literature (Harvey 2021). Cohens Kappa was not used as the data were not nominal (DATAtab 2024). An ICC was derived from the application of ANOVA in Microsoft Excel® (Bobbitt 2023).

Using the anonymous code, responses from participants that had completed the OS twice were identified and analysed for intra-observer agreement. Only paired responses completed according to the study design (outlined above) were included in this analysis. Where there were missing values, the question for that observer was removed. Using the observers’ anonymous codes, the number of different observers could be determined. The agreement between ratings of each observer was analysed to test the inter-observer reliability. The first of the paired tests and all the single tests (where observers only completed the test once) were used for agreement analysis. Tests with missing values were removed prior to analysis.

## Results

### AWRAT trial

Eighty-three percent of participants were animal welfare officers/veterinarians and there were two biosecurity officers, one animal health officer, one police officer and another not defined. Thirty-five participants completed at least one animal welfare risk assessment using the AWRAT over the study period.

### Trial data-set

The trial data-set included 67 entries. The adjusted R was higher when the linear regression just included the assessments with MOC of ≥ 90 and = 100 (Table 2). When only assessments with a MOC =100 were included the adjusted R was 0.835 compared with 0.672 when all data were included, regardless of the MOC. This shows that when all factors in the AWRAT were given a rating, the AWRAT predicts 83.5% of the variation in the risk to welfare as measured by the officer’s overall subjective assessment (WR). All tests were significant (*P* ≤ 0.05). This suggested that, in the trial data-set, the AWRAT is better at measuring the risk of poor welfare when more factors were included in the assessment.

**Table 2.** Linear regression of trial data set, comparing the ability of the AWRAT-RR to identify livestock at risk of poor welfare using all the data and only those assessments with MOC ≥ 70, ≥ 80, ≥ 90 and 100

Trial data-set	Regression statistics				Intercept			AWRAT-RR		
Data analysed (MOC)	Multiple R	R Square	Adjusted R Square	Standard Error	Coefficients	Standard Error	*P*-value	Coefficients	Standard Error	*P*-value
All data	0.820	0.672	0.667	17.320	4.501	4.864	0.358	0.967	0.084	2.17E–17
≥ 70	0.824	0.678	0.672	17.456	3.553	5.128	0.491	3.553	5.128	2.09E–15
≥ 80	0.819	0.670	0.663	17.993	3.470	5.517	0.532	0.929	0.096	1.18E–12
≥ 90	0.884	0.781	0.774	15.778	1.069	5.221	0.839	0.988	0.091	2.02E–12
=100	0.914	0.835	0.822	13.530	5.326	6.396	0.420	0.909	0.112	1.91E–06

MOC-measure of completeness

AWRAT-RR- Animal Welfare Risk Assessment Tool- Risk Rating

### Test data-set

For the test data-set, the adjusted R values were higher overall and varied less than the trial data-set. There was no increased ability to identify livestock at risk of poor welfare as the MOC increased in the test data-set analysis. The slightly increased ability of the AWRAT to predict the variation in risk of poor welfare in the test data-set may be due to increased familiarity with the assessment process by participants and some improved self-calibration. The reliability of the assessment with variation in MOC is a positive finding for the tool, demonstrating its flexibility and reliability for use in situations where not all of the factors can be rated. All regression analyses were significant (*P* ≤ 0.05). The adjusted R when all the data (regardless of the MOC) were included in the AWRAT-RR was 0.810 compared with 0.809 when only assessment with a MOC = 100 were included (Table 3).

**Table 3.** Linear regression of test data-set. Determining the ability of the AWRAT-RR to identify livestock at risk of poor welfare using all the data and only those assessment with MOC ≥ 70, ≥ 80, ≥ 90 and 100

Test data-set	Regression statistics				Intercept			AWRAT-RR		
Data analysed (MOC)	Multiple R	R Square	Adjusted R Square	Standard Error	Coefficients	Standard Error	*P*-value	Coefficients	Standard Error	*P*-value
All data	0.900	0.810	0.805	14.335	3.425	5.191	0.513	0.947	0.075	6.63E–15
≥ 70	0.893	0.797	0.791	15.234	3.210	5.676	0.576	0.937	0.084	1.25E–12
≥ 80	0.897	0.805	0.798	15.340	2.421	5.831	0.681	0.930	0.086	1.86E–11
≥ 90	0.900	0.810	0.803	3.267	5.862	0.582	0.582	0.916	0.087	7.10E–11
=100	0.899	0.809	0.793	16.722	3.173	7.936	0.696	0.919	0.129	1.22E–05

MOC-measure of completeness

AWRAT-RR- Animal Welfare Risk Assessment Tool- Risk Rating

### Observation study

Twenty-three participants completed the OS twice, according to the study protocol. Seventeen of the observers had zero missing values, three had one missing, two had three missing and one, five missing values. The intra-observer reliability was good. Eighty-seven percent of observers (n = 20) had an ICC of ≥ 0.8, and the remainder (n = 3) between 0.68–0.79. There were 45 participants who completed the observation test at least once, but only 29 had zero missing values and were analysed for inter-observer reliability. The inter-observer reliability agreement was moderate with an ICC of 0.67.

## Discussion

An Animal Welfare Risk Assessment Tool (AWRAT) was developed previously by the authors, based on risk factors more commonly observed on properties where poor livestock welfare had been identified (see Williams *et al.* 2024a, published in conjunction with this paper). Risk factors were included in the AWRAT if they were relatively easy to observe during farm visits, relevant to extensive production systems all over Australia and rated by industry as being significantly more/less relevant on farms where the welfare of the animals was low compared to those with good welfare. Lastly, only risk factors that had a logical connection to the welfare of the livestock were included. For example, factors relating to the suitability of nutrition and treatment, rather than farms with a red mailbox. As was shown in this study, the tool needed to be robust enough to determine a measure of risk without necessarily having a rating for every factor, as it was not always possible to observe all factors on every farm visit. Initial testing indicates that the AWRAT is able to identify farms where the overall welfare state of the animals is poor. Further research is needed to verify its reliability at identifying livestock at risk of poor welfare over a longer period.

### The AWRAT trial

The AWRAT-RR was effective at identifying farms with livestock showing poor welfare even when the MOC was 70. This provides the tool with flexibility to still be useful when not all the factors are observable during a farm visit. For example, on a farm where there is a failure to shear, the inspecting officers may not have the opportunity to assess all of the factors regarding nutrition since not enough of the farm is seen to be able to make those assessments. However, providing ratings for all factors would be encouraged in all assessments because this would provide a more complete record of all the areas that need improvement. This would also provide a good record to which later assessments could be compared.

As noted by Williams *et al.* (2024b), in Australia, 27% of people found with livestock welfare non-compliance subsequently reoffended. Reoffending was included as an additional factor in an early version of the AWRAT algorithm, but it overwhelmed the model, making the other factors significantly worse predictors of poor welfare. If the AWRAT has the capacity to identify livestock that are at risk of poor welfare this, by extension, provides a measure of the likelihood of a property reoffending. For example, if a farmer is currently or has previously been found to have livestock welfare non-compliance and an AWRAT assessment indicates that the livestock are at risk of poor welfare, this would suggest an increased likelihood of reoffending (given that they have offended already). The ability to identify farms where there is a high risk of reoffending would obviously be advantageous from an animal welfare perspective. In addition, if intervention could prevent a repeated offence, there would be less resources required to manage the situation as well as lessening the personal and financial impacts on the farmers themselves. A longitudinal study is needed to determine the AWRAT’s ability to identify reoffenders. In such a study, an AWRAT assessment would be completed at the first and last visit of the initial investigation. Then, over several years, investigating officers would be asked to report any recidivism. When reoffending occurs, the relationship to the initial AWRAT assessments could be analysed using correlation with the earlier risk assessments. In the current study, investigations that involved reoffenders were identified but there was no AWRAT from previous investigation(s) to analyse.

Initial testing in this study has shown the AWRAT has an ability to identify farms where the overall welfare of the livestock is poor, when compared to a single, overall WR. Further testing of the AWRAT is needed before it can be considered for field use. To mimic normal operations, participants in further trials would complete training prior to use of the tool. This would serve to optimise field observations and calibrate assessments. Participants would then be asked to complete an AWRAT after every farm visit and then the resulting AWRAT-RR could be evaluated qualitatively, through discussion with the attending officer(s) considering prior offences, the number of animals affected, and the severity of the issues. Secondly, using quantitative methods, a longitudinal study (as previously mentioned) could determine the AWRAT’s ability to identify livestock at risk of poor welfare based on recidivism over time. Further work is required before the tool may be used to inform sentencing in court.

As the AWRAT is based on factors that are easy to identify and do not require advanced skills to assess, an independent person could potentially be engaged to complete an assessment to verify an officer’s assessment. This might be particularly relevant for cases in which seizure of the animals or prosecution are likely to occur. If the complaint is substantiated and a breach in the legislation is identified, the risk assessment could be used to guide the planned response. As mentioned previously, this could assist in planning the intensity of the visits, allocation of resources and use of the legislation to assist with resolution. Potentially, the tool may also be used by extension staff, stock agents and veterinarians as a way of identifying properties at risk of poor welfare early, facilitating timely intervention. By identifying livestock at risk of poor welfare it may enable improved and more targeted intervention and, by extension, hastening changes in attitude and management that can result in more sustained resolution of livestock welfare issues. Potentially the tool could be used by farmers themselves to estimate the risk of poor welfare given the circumstances on their own farms. In domestic violence risk assessment, the victim also completes their own assessment of risk (ACT Government 2022).

It is proposed that ultimately the AWRAT-RR could provide a measure of risk according to a number of categories, for example, low, medium and high risk. This is a risk assessment, and there is no certainty about a risk assessment (BJA 2023), but it provides informed guidance to assist in decision-making. There will be farmers that have a high risk of poor welfare, that never have poor welfare and, conversely, those with low-risk ratings may be found to have livestock with poor welfare. The AWRAT is a tool, and any welfare investigation must also be supported by thinking. In some risk assessment tools used in other disciplines there is also a ‘practitioner professional judgment’ component, ensuring that there is ongoing thinking, outside of the risk factor-based assessment (ACT Government 2022). Considering the findings from this study, and the potential additional application of the AWRAT as a guide to the improvement measures that are required, it is anticipated that for an AWRAT-RR to be considered valid, it will require a minimum of at least one rating in each topic area and MOC of at least 70.

### The observation study

The OS found observation, in the context of features on a farm, to be a reliable tool. The intra-observer reliability was good and the inter-observer reliability moderate. This is important as the ability of the AWRAT to identify livestock at risk of poor welfare relies upon consistency of observation between individuals and on different occasions with the same individual. It is also likely that observation skills can be improved with training (Travnik *et al.* 2022; de Wilde *et al.* 2023).

Observational reliability is ideally assessed in the exact context in which the observation tool was intended to be used (Pfeifer *et al.* 2019), for example, on-farm. As this was a national study, it was not economically or logistically feasible to have all participants complete an assessment on the same farm at the same time. Although translating the findings from an experimental setting to that in the field can be challenging, photographs and videos have been suggested as an alternative method to assess observer reliability (Martin & Bateson 1986) as cited by Pfeifer *et al.* (2019). Most of the factors in the AWRAT were easily represented as a fixed image assessment in the OS. It is acknowledged that during normal farm inspections, features such as fences and feed availability would be assessed on a whole farm basis, rather than a single static photograph. Variability in observations between stakeholders has been reported previously (Duijvesteijn *et al.* 2014), with greater reliability from those with experience (de Wilde *et al.* 2023). While others have found trained assessors and those with different experience to be reliable assessors (Diaz-Lundahl *et al.* 2019).

### Study challenges and limitations

One of the biggest challenges with developing a risk assessment tool for poor livestock welfare is that there is no gold standard measure against which to compare. In this study, participants were asked to provide a WR prior to the AWRAT assessment to provide a basis for comparison. This was not ideal, as the comparison was made between two novel measures provided by the same participant. To determine the WR, participants were asked to consider the ‘*overall welfare status of the livestock on the farm’* during their visit. In contrast, the AWRAT included assessment of a number of specific factors about the farm, facilities, nutrition and management. Typically, farm welfare investigations focus upon determining whether there has been a breach of the legislation and, if so, collecting evidence to demonstrate that. Participants in this study had experience in animal welfare investigations and were accustomed to making a judgment on the welfare state of the livestock during a farm visit. Whilst, typically, the welfare state of the animals would not be quantified by a specific value during investigations, detailed aspects of their health and welfare would be considered and recorded qualitatively. It was likely, therefore, to only be a simple adjustment for participants to provide an overall welfare status after a farm visit. Given the common source of the WR and AWRAT assessment, the ability for one value to predict the other with linear regression was expected to some extent. The WR provided a good approximation of the welfare status of the animals at a point in time. The WR however does not consider the ongoing risk of poor welfare, which is a key advantage of using the AWRAT. In addition, as the AWRAT requires the user to consider a number of different aspects of the farm, it provides a framework for systematic observation and assessment of risk. This is likely to improve transparency of the assessment of risk, provide a point of comparison and improve consistency between officers.

The differing seasons in which the data were collected for analysis in trial and test data-sets will have likely impacted the state of certain aspects of the farm, particularly the amount of feed available which is one factor in the AWRAT. It is not expected that the season would have made a significant difference to the AWRAT assessments since the majority of the factors are not based on factors that show seasonal variation. The AWRAT algorithm also makes allowances for assessments where not every factor has a rating. Therefore, if there was a difference in the number of factors with ratings between seasons this would be reflected in the MOC for the assessment. This is important because the tool needs to be reliable at making welfare assessments regardless of the season.

The limitations of this study included the use of the WR to compare the AWRAT assessment, which was discussed earlier. Secondly, only slightly over half of participants completed the observer test twice, no less than ten days apart. This is a disappointing but not surprising level of engagement despite a number of reminder emails. This study relied upon participation based on goodwill, without an incentive provided. In the initial briefing, many participants flagged that they were time poor, and this might have been a contributing factor to the meagre level of repeated tests.

### Animal welfare implications

The ability to identify livestock at risk of poor welfare using an animal welfare risk assessment tool (AWRAT) could enable improved livestock welfare outcomes on-farm. In situations where poor livestock welfare had been identified, the assessment would provide a structured way to assess and reassess the situation. In addition, the AWRAT is likely to improve consistency with assessment between individuals. Furthermore, the assessment would provide a structured way to assess changes to the situation over time. In scenarios where an issue of welfare non-compliance is resolved, the assessment could identify livestock that remain at risk of poor welfare, and this may indicate the likelihood of reoffending. Identifying livestock at risk of poor welfare early would facilitate intervention, extension, and education to improve the care and welfare of the livestock. Potentially, an AWRAT may provide a structured format to discuss with the farmer what needs to be improved and provide a way to monitor those improvements. In court, an AWRAT assessment might be used to inform the judge of the likelihood of reoffending in cases prosecuted under animal welfare legislation, and this might inform sentencing.

## Conclusion

The AWRAT has shown potential in identifying farms with non-dairy cattle, sheep and goats at risk with poor welfare in extensive farming systems in Australia. The structured framework of the AWRAT is likely to improve the breadth and consistency of observations during livestock welfare inspections. This will also enable factors that are not typically part of a welfare assessment but possible contributors to improper care to be considered, measured and monitored. Observation is the key measurement tool for the AWRAT, and the intra- and inter-observer reliability were good and moderate, respectively. The practical nature of the factors in the AWRAT make it a suitable framework to guide the necessary changes to decrease the risk and maintain welfare standards. The AWRAT is now ready to trial in the field for extensively grazed, non-dairy cattle sheep and goats.

## Supporting information

Williams et al. supplementary material Williams et al. supplementary material

## Data Availability

The data-set presented in this paper are not readily available because our ethics approval specifies ‘*Data are aggregated and analysed and reported as a group, therefore no findings that could identify any individual will be published’.* Therefore, we cannot supply raw data even though they are anonymised, without special approval from the ethics committee through an amendment. Requests to access the data-sets should be directed to NW at natscottw@gmail.com.

## Supplementary material

The supplementary material for this article can be found at http://doi.org/10.1017/awf.2024.28.

## References

[r1] ACT Government 2022 *ACT Domestic and Family Violence Risk Assessment and Managment Framework. Supporting Integrated Domestic and Family Violence Service System.* ACT Government: Canberra, ACT, Australia. https://www.communityservices.act.gov.au/__data/assets/pdf_file/0010/2035675/ACT-Gov-Domestic-and-Family-Violence-RAMF.pdf (accessed 15 March 2024).

[r2] Bateson M and Martin P 2021 Measuring Behaviour: An Introductory Guide, Fourth Edition. Cambridge University Press: New York, NY, USA.

[r3] Bureau of Justice Assistance (BJA) 2023 *What Is Risk Assessment.* https://bja.ojp.gov/program/psrac/basics/what-is-risk-assessment#:~:text=Understanding%20Risk%20Assessment&text=First%2C%20risk%20assessments%20provide%20a,a%20person’s%20behavior%20with%20certainty (accessed 25 April 2023).

[r4] Bobbitt Z 2023 How to Calculate Intraclass Correlation Coefficient in Excel. https://www.statology.org/ (accessed 19 February 2024).

[r6] Commons C 2023 Creative Commons Legal Code. *Attribution-ShareAlike 2.0.* Creative Commons. https://creativecommons.org/licenses/by-sa/2.0/legalcode (accessed 5 May 2024).

[r7] DATAtab 2024 *Cohen’s Kappa.* https://datatab.net/tutorial/cohens-kappa (accessed 19 February 2024).

[r8] DAWE 2019 *Animal welfare in Australia.* https://www.agriculture.gov.au/animal/welfare/animal-welfare-in-australia#animal-industry-groups (accessed 15 August 2020).

[r9] DEECA 2023 *Report animal cruelty.* https://agriculture.vic.gov.au/livestock-and-animals/livestock-health-and-welfare/report-animal-cruelty (accessed 14 July 2023).

[r10] Desmarais SL, Nicholls TL, Wilson CM and Brink J 2012 Using dynamic risk and protective factors to predict inpatient aggression: Reliability and validity of START assessments. Psychological Assessment 24(3): 685–700. 10.1037/a0026668 22250595 PMC3470450

[r11] de Wilde B, Joosten F, Venderink W, Davidse MEJ, Geurts J, Kruijt H, Vermeulen A, Martens B, Schyns MVP, Huige JCBM, de Boer MC, Tonino BAR, Zandvoort HJA, Lammert K, Parviainen H, Vuorinen A, Syväranta S, Vogels RRM, Prins W, Coppola A, Bossa N, ten Broek RPG and Huisman H 2023 Inter- and intra-observer variability and the effect of experience in Cine-MRI for adhesion detection. Journal of Imaging 9(3): 55. 10.3390/jimaging9030055 36976106 PMC10054690

[r12] Diaz-Lundahl S, Hellestveit S, Stubsjøen SM, Phythian CJ, Moe RO and Muri K 2019 Intra- and inter-observer reliability of Qualitative Behaviour Assessments of housed sheep in Norway. Animals 9(8): 569. 10.3390/ani9080569 31426493 PMC6719082

[r13] Duijvesteijn N, Benard M, Reimert I and Camerlink I 2014 Same pig, different conclusions: Stakeholders differ in qualitative behaviour assessment. Journal of Agricultural and Environmental Ethics 27(6): 1019–1047. 10.1007/s10806-014-9513-z

[r14] FAWAC 2018 *Early Warning/Intervention System (EWS).* http://www.fawac.ie/earlywarningsystemews/ (accessed 23 July 2022).

[r15] Foundation W 2023 *Search media.* https://commons.wikimedia.org/w/index.php?search=sheep&title=Special:MediaSearch&go=Go&type=image (accessed 22 September 2022).

[r16] Garrett B and Monahan J 2019 Assessing risk: The use of risk assessment in sentencing. Judicature 103.

[r17] Harrell FE 2022 Analysis of observer variability and measurement agreement. In: Harrell FE and Slaughter J (Eds.) Biostatistcs for Biomedical Research. Department of Biostatistics, Vanderbilt University School of Medicine: Nashville, USA.

[r18] Harvey N 2021 *A Simple Guide to Inter-rater, Intra-rater and Test-retest Reliability for Animal Behaviour Studies.* https://osf.io/preprints/osf/8stpy (accessed 7 May 2024).

[r19] Hedman F, Hultgren J, Röcklinsberg H, Wahlberg B and Berg C 2018 Non-compliance and follow-up in Swedish official and private animal welfare control of dairy cows. Animals 8(5): 72. 10.3390/ani8050072 29738491 PMC5981283

[r20] Kleiman M, Ostrom BJ and Cheesman FL 2007 Using risk assessment to inform sentencing decisions for non-violent offenders in Virginia. Crime & Delinquency 53(1): 106–132. 10.1177/0011128706294442

[r21] Martin P and Bateson P 1986 Measuring Behaviour: An Introductory Guide. Cambridge University Press: New York, NY, USA.

[r22] Mercer A, Lau A and Kennedy C 2018 *How different weighting methods work.* https://www.pewresearch.org/methods/2018/01/26/how-different-weighting-methods-work/ (accessed 26 August 2023).

[r23] Morton R, Hebart ML and Whittaker AL 2018 Increasing maximum penalties for animal welfare offences in South Australia: Has it caused penal change? *Animals (Basel)* 8(12): 10.3390/ani8120236 PMC6316723 30544781

[r24] Morton R, Hebart ML and Whittaker AL 2020 Explaining the gap between the ambitious goals and practical reality of animal welfare law enforcement: A review of the enforcement gap in Australia. Animals (Basel) 10(3). 10.3390/ani10030482 PMC7142490 32183062

[r25] O’Connell ME, Boat T and Warner KE 2009 Preventing Mental, Emotional, and Behavioural Disorders Among Young People. The National Academies Press: Washington, DC, USA. 20662125

[r26] Pfeifer M, Eggemann L, Kransmann J, Schmitt AO and Hessel EF 2019 Inter- and intra-observer reliability of animal welfare indicators for the on-farm self-assessment of fattening pigs. Animal 13(8): 1712–1720. 10.1017/S1751731118003701 30630538

[r27] Popović ZB and Thomas JD 2017 Assessing observer variability: a user’s guide. Cardiovascular Diagnostic Therapy 7(3): 317–324. 10.21037/cdt.2017.03.12 PMC5440257 28567357

[r28] RSPCA 2022 *Our role in enforcing the law.* https://www.rspca.org.au/what-we-do/our-role-enforcing-law (accessed 4 December 2022).

[r29] SAC 2019 Animal Cruelty Offences in Victoria. Sentencing Advisory Council: Melbourne, VIC, Australia. https://www.sentencingcouncil.vic.gov.au/sites/default/files/2019-08/Animal_Cruelty_Offences_in_Victoria.pdf (accessed 15 March 2024).

[r30] Sandgren CH, Lindberg A and Keeling L 2009 Using a national database to identify herds with poor welfare. Animal Welfare 18: 523–532. 10.1017/S0962728600000944

[r31] Schooling CM and Jones HE 2018 Clarifying questions about ‘risk factors’: Predictors versus explanation. Emerging Themes in Epidemiology 15(1): 10. 10.1186/s12982-018-0080-z 30116285 PMC6083579

[r32] Temple D and Manteca X 2020 Animal welfare in extensive production systems is still an area of concern. Frontiers in Sustainable Food Systems 4(154). 10.3389/fsufs.2020.545902

[r33] Travnik IC, Machado DS and Sant’Anna AC 2022 Do you see the same cat that I see? Inter- and intra-observer reliability for Qualitative Behaviour Assessment as temperament indicator in domestic cats. Animal Welfare 31(3): 319–327. 10.7120/09627286.31.3.004

[r34] Väärikkälä S, Koskela T, Hänninen L and Nevas M 2020 Evaluation of criminal sanctions concerning violations of cattle and pig welfare. Animals 10: 715. 10.3390/ani10040715 32325918 PMC7222770

[r35] van Ginneken EFJC 2019 The use of risk assessment in sentencing. In: de Keijser JW, Roberts JV and Ryberg J (Eds.) Predictive Sentencing: Normative and Empirical Perspectives pp 9–32. Hart Publishing: Oxford, UK.

[r36] Victoria State Government 1986 *The Prevention of Cruelty to Animals Act. Authorised version No.* 095. Victoria State Government: VIC, Australia.

[r38] Williams N 2024 What are the triggers, challenges, attitudes and behaviours that contribute to poor welfare of livestock? Can these situations where livestock are at risk of poor welfare be predicted? Ph.D. Thesis, University of Melbourne. https://hdl.handle.net/11343/344670

[r39] Williams N, Chaplin S, Hemsworth L, Shephard R and Fisher A 2024a Analysis of substantiated welfare investigations in extensive farming systems in Victoria, Australia. Australian Veterinary Journal. 10.1111/avj.13342 38798110

[r40] Williams N, Hemsworth L, Chaplin S, Shephard R and Fisher A 2024b Analysis of livestock welfare investigations in extensive farming systems, in prep. 10.1111/avj.13342 38798110

[r41] Zsidisin GA, Ellram LM, Carter JR and Cavinato JL 2004 An analysis of supply risk assessment techniques. International Journal of Physical Distribution & Logistics Management 34(5): 397–413. 10.1108/09600030410545445

