# Supplementary material for: Can an animal welfare risk assessment tool identify livestock at risk of poor welfare outcomes?
Source: Anim Welf. 2024 Sep 16;33:e32. doi: 10.1017/awf.2024.28 (PMC11418070; doi:10.1017/awf.2024.28)
Supplement: Williams et al. supplementary material [file S0962728624000289sup001.pdf]

Supplementary material

Standard operating procedure – DRAFT Animal Welfare Risk Assessment Tool (AWRAT-D) project

In the information material provided to participants during this research the AWRAT was called the DRAFT-AWRAT (AWRAT-D). However, that was changed to the AWRAT in the preparation of this manuscript.

Scope for use of AWRAT-D

The AWRAT is **ONLY** suitable to be used:

- By participants that are 18 years old or older and have at least 6 months experience with livestock
- In situations where the livestock involved are NON-DAIRY: cattle, sheep or goats
- On extensive farming systems

The AWRAT is **NOT** designed for:

- Intensive farming systems
- Dairy livestock
- Measuring the severity of a welfare situation
- Sharing with the farmer/ owner

Methodology

While the tool is very easy to use, it is important that the assessment is completed accurately and without bias to ensure meaningful outcomes from this study.

The following points are vital:

- **After** every farm visit where there are livestock (NON-DAIRY: cattle, sheep or goats) complete an AWRAT-D assessment, please.
- Farms should have at least 10 livestock on a property in an extensive production system. This might include a mixture of non-dairy: cattle, sheep and goats. There may be other agriculture enterprises on the property as well, for example, cropping, forestry, pig or poultry farming.
- This includes all revisits, even if it is several times over the study period or just 2 days after the last visit (this will allow the repeatability of the test to be compared)
- Farm visits may be welfare and non-welfare related. Examples of non-welfare visits might include: audits, disease investigations, other project related inspections
- Use the link provided to access the AWRAT-D on Qualtrics. Note: a blank copy of the AWRAT-D will open every time you follow the link or QR code, ready for a new assessment.

- All eligible participants attending the farm visit are asked to please complete their own AWRAT-D assessment for that property. The number of people present will vary depending on the organisation / business and nature of the visit. When there is more than one person attending please ensure all participants complete the assessment individually without discussion with others.
- Provide a value for all factors in the assessment that you have been able to verify, ideally all factors will have a value. You may not strictly observe the factor but have gathered from previous interactions or deduced a value based on other information made available to you. For e.g. if cows, heifers and calves of all ages are in the same herd, they are not being fed according to their productive status, as this can't be done when they are mixed together.
- The tool **does not** require a value for every factor however, in order to make a risk assessment. If you are not sure about a factor then please mark the 'unable to verify box' in the tool
- Avoid making assumptions or guessing
- **Do NOT** consult with the farmer to complete the assessment
- **Do NOT** travel around the farm any more than would be usually required for the visit
- Complete the assessment by yourself without comparing or consulting with others in your team. The study aims to compare how similarly different participants rate the same situation.
- Assign a code to each visit so that repeat visits to the same farm can be identified for comparison, while still ensuring that the farm location is still not identifiable.

<u>Code questions</u>

First letter of your middle name (or if not applicable, surname)

First letter of your favourite sports team (e.g. AFL, league, netball, basketball)

First 2 letters of location (town) where farm is located- ____ ____

First letter of farmers surname- ____ (if unknown put $)

Todays date ___ ___/___ ___/ ___ ___ ___ ___

AWRAT-D

Sections A and B

These are additional questions just for this study, this information will allow comparison between responses most accurately, only 6 questions in total.

Section C: AWRAT-D

To be completed once you have left the farm

There are 20 questions in the AWRAT-D. The first 18 are to be answered by applying a value using a slider scale, according to how relevant you think that factors is on the farm you have just visited. The last two are tick box answers.

The approach should be an informed judgment based on what you have seen and what you know about the farm. It needs to be an overall impression. For example, the first question is about fencing, if the majority of the fencing is great but there is a small area, or a single paddock with poor fencing then overall the fencing is pretty good, but not excellent. It may be that the paddock with poor fencing is a new recently purchased paddock or a paddock the farmer is upgrading next.

I have provided some detail about each factor below to clarify what it means in the context of the AWRAT-D.

Not true*___________|________________ *Very true

I don't know / I am unable to verify this ☐

Farm

1. ***There is evidence that the farm fencing is of an effective standard (e.g. stock proof, swinging gates)***

Indirect observations

- Livestock out on the road may indicate poor fencing (this often forms part of the initial complaint… 'the animals are always out on the road')
- Livestock are run as a single mob, possibly because the fences are not good enough to keep them divided into separate mobs/ herds.
- Males (bulls, rams or bucks) are run with the mob / herd all the time because the fences are not good enough to keep them apart. Although this might be because the farmer doesn't remove the males.
- Failing to wean also may reflect poor fencing, as they cannot be adequately separated.
- Complaints about lice may arise due to concern about animals with lice getting thought fences

- Animals caught up in wire: occurs more often when fences are poor and livestock walk over them as they move around the farm
- Extensive use of electric fencing (particularly on farms with cattle) can be an indication that the fences are poor. But not always.

Direct observations

- Overall the fences are largely in disrepair. Fences post broken or leaning over, wire, sagging or on the ground
- Loose wires, ring lock that has been squashed down
- Gates that don’t swing, generally more than one
- Lots of patched up areas of fencing using odd bits of wire, hay band (bindertwine), tin

2. ***There is evidence that the stock handling facilities are suitable and in working order***

Indirect observations

- Sheep or goats that have not been shorn or crutched in a timely manner, may indicate a lack of facilities, shearing shed and / or yards
- Failure to preform preventative measure or treatment for fly strike may result from a lack of suitable yards and / or shearing shed.
- Animal that have not been appropriately weaned because a lack of suitable yards
- Animals that have not been marked in a timely manner or appropriate manner.
- Properties where there are a lot of mikey bulls (young uncastrated males approximately 12 - 18 months old or more) may have inadequate yards for marking, drafting or selling large strong and potentially aggressive males
- Delay in selling stock routinely can occur when there are no facilities, or the facilities are in poor condition and challenging to use, particularly for cattle
- Poor access to yards by trucks, due to weather affected tracks can limit selling opportunities of livestock

Direct observations

- There is no suitable shearing facilities on a property where there are sheep or goats that require shearing
- There are yards, but they are clearly dilapidated
- There are no suitable yards for the species present, e.g. sheep yards on a farm with cattle

3. ***There is evidence that the feed and water areas and facilities are clean, functional and accessible to all livestock***

Indirect observations

- When one class of animal is affected by hunger or thirst in a single mob/ herd may indicate that they do not have access to adequate feed or water, e.g. tall water troughs can exclude access from young animals
- An increase in incidence in faecally transmitted diseases, such as coccidiosis, can occur when the supplementary feeding and watering areas are wet with high amounts of faecal contamination

Direct observations

- Non-permanent water access, such as troughs that need manually filling by the farmer are a higher risk of becoming dry.
- Water or feed troughs are really dirty e.g. Lots of faecal contamination or dirt
- The water present is not suitable, for e.g. dirty, contaminated with carcasses, salty
- The trough or dam is surrounded by a muddy area, that is a bog risk, especially for very young or weak stock
- The bank leading done to the water is very steep making access difficult
- The water source is not permanent and there are times when the trough is empty
- The troughs are too high for young stock to reach in to drink or eat
- The feeding or water troughs are not suitable for stock, they pose a risk of injury, no not provide suitable safe access
- On occasion, electric fencing can contact troughs to make them 'live' resulting in animals not drinking from them
- Grain and pellets are fed in a trough or equivalent, so livestock are not eating off the ground
- Animals bogged in mud around water sources may indicate that the water supply is leaking. Cattle can damage water supply pipes by rubbing at them of standing on them

4. ***There is evidence that the farm has an equivalent amount of pasture (or better) to that on surrounding farms***

<u>Direct observations</u>

- On the drive to the farm you will get a sense of the amount of pasture available in the area and on neighbouring or near by properties. This provides a sense of what is a reasonable / average amount of feed that could be expected given the current seasonal conditions in that area.

5. ***There is evidence that paddocks with livestock are suitable to avoid illness and accidental injury (e.g. toxic plants, loose wire, metal, old batteries)***

Indirect observations

- If there are animals present with injuries such as cuts and wire caught around their legs, it would suggest they have been caused by an unsafe environment
- An increase in incidence of deaths and unwell livestock can be caused by access to toxic plants. This might be more likely when there is shortage of pasture and the animals are hungry and more inclined to eat plants that they would otherwise normally avoid.

Direct observations

- Toxic plants are more likely to become established in pastures that are overgrazed
- Purchase and feeding of poor quality (but cheaper) hay can introduce weeds to a farm.
- Livestock do not have access to old tips
- Livestock do not have access to sheds, stored chemicals, old dips
- There is no rubbish in the paddock for example: garden waste, car bodies, old fencing materials, chemical drums

6. ***There is evidence that dead animals are removed from paddocks***

Direct observations

- The presence of carcasses at different locations across the farm, particularly at different stages of decomposition would suggest that removal of carcasses is not routinely performed on the property.
- It is not uncommon for this to be included in a welfare complaint, 'there are carcasses in the paddock and the farmer never removes them'
- If you are aware that the farm has experience losses, and none can be observed, then it is likely they are routinely removed.
- Evidence of a pile of carcasses somewhere on the farm, some of which are relatively recent, suggests that the farmer removes carcasses from the paddock.

Nutrition

7. ***There is evidence that livestock are fed according to their reproductive state (e.g. empty, pregnant or lactating)***

Indirect observations

- When animals of different reproductive states are in the same mob or herd they cannot be fed according to their productive state, e.g. livestock in late and early pregnancy, livestock that are lactating and those that are dry
- When the males (ram, bull or buck) are with the mob / herd all year around, livestock cannot be fed according to their reproductive states

Direct observations

- Stock are in groups according to their reproductive state
- There is evidence of supplementary feeding to groups that have a higher metabolic requirement

8. ***There is evidence that some paddocks have good feed remaining***

Direct observations

- There is suitable pasture remaining in some paddocks which is appropriate for the species of livestock present on the farm.

9. ***There is evidence that feed offered is suitable for the class of animal (e.g. energy, protein etc)***

Direct observation

- When the mob / herd consists of animals of different productive states and one class of animals are poor, it suggests that the nutrition available does not meet their productive requirements. Typically, this would include heavily lactating females.
- On occasion, a small number of animals in a mob or herd maybe in poor condition, this may reflect an individual animals' inability to make use of the feed available. This could be due to disease such as Johnes' disease, cancer or worn teeth. Food on offer maybe suitable in these cases but there has been a failure to treat or euthanise animals that are unable to maintain their weight.
- When the vast majority of a herd/ mob are in suitable body condition, it can be assumed that their diet is adequate. This might include a combination of pasture and supplementation or just pasture or just supplementary feed during drought conditions.
- When the majority of a mob / herd are in poor condition, they are not being provided with adequate quality and /or quantity of feed. This might be complicated by diseases such as parasites or Johnes disease within the mob / herd. The presence/ absence of adequate pasture in the paddocks and evidence of supplementary feed can assist in determining this factor

10. ***There is evidence that the supplementary feeding is offered before significant weight loss occurs***

Direct observations

- When livestock are in good condition and on a bare paddock, it can be assumed that they have been supplementary fed prior to excessive weight loss. Alternatively, animals in good condition on a bare paddock may have only just have been put in the paddock recently, without the opportunity yet, for significant weight loss.

11. ***There is evidence that there is some supplementary feed remaining in the paddock after the livestock have finished feeding***

Direct observation

- In a paddock where livestock have been supplementary fed recently, the livestock may have moved away from the feed and stopped eating (suggesting they have had enough to eat) and there is supplementary food remaining. This factor is **NOT** counted as true if the feed that is remaining is very poor quality, such as straw, or weather affected hay, as this is largely unpalatable and does not reflect a situation where there is more feed supplied than the livestock can eat.

12. ***There is evidence that all animals have equal access to supplementary feeding (e.g. well spread out or continuous access)***

Indirect observations

- When there is a marked difference in the condition of type of livestock within a mob / herd it may reflect a situation where there is a non-equal access to supplementary feed. For example, young animals may not compete well to access supplementation when it is only spread over a small area and they maybe in lighter condition
- When there are some adult animals in the same reproductive state that are in good condition while others are poor condition, this can reflect unequal access to feed when bullies get to the feed first
- When access to supplementation is not equal, bossy / dominant animals may push the more submissive animals away to gain preferential access to the feed. This can result in the dominant animals being in good condition while the submissive animals are in poor condition.

Direct observations

- With intermittent supplementary feeding e.g alternate day feeding, equal access is most relevant, as livestock are generally hungry and eager to access the feed all at once. In such cases hay should be spread out in a line long enough that all the livestock can access it at once. Similarly, for grain or concentrates equal opportunity should be offered, be making an extended link of feed.
- Hay available in a hay feeder is suitable when there is continuous access.
- Grain or concentrates available ad lib through a feeder also is suitable, as long as access is continuous

Management and nutrition

13. ***There is evidence that the lambs, calves and / or kids within a mob / herd are all a similar age (within 2-3 months of each other)***

Indirect observations

- In all year around breeding systems, offspring will not all be of similar age

Direct observations

- Offspring vary in age considerably, this might include from newborns to weaning age and some at the age of first joining and other on the point of parturition. This is more obvious when males are with the females all year round with no controlled joining time. Offspring will vary from semi adult to new born

14. ***There is evidence that animals with conditions that are unresponsive or not economical to treat are humanely culled without delay (e.g. lameness, cancers, chronic scouring, congenital abnormalities)***
15. ***There is evidence that the investigation and treatment of health conditions affecting livestock are completed as soon as the problem is identified***

The observations for factor 14 and 15 are very similar and have therefore been included together here. For question 14 the animals are more severely affected, and euthanasia is likely to be the best and most humane outcome.

Direct observations

- Animals that have evidence of chronic illness might include:
    - Individual animals that are in very poor body condition compared to the rest of the mob / herd.
    - Animals that have evidence of fly strike, where the size of the maggots is evidence that infection has been going for more than a few days
    - Fly strike where the affected area is extensive, indicating that problem has been going on for more than a few days
    - Animals that have lameness and associated muscle atrophy of the affected limb / s
    - Livestock with obvious growths / cancers
    - Livestock that are recumbent and there is a large amount of faecal material build up behind them suggests that the animals has been down for some time
    - Livestock that are recumbent and have a large amount of dirt scratched away from paddling, or trying to get up
    - Livestock with genital malformations that are weeks to months of age (these are only relevant if they are interfering with the animal quality of life)
    - Livestock with evidence of chronic scouring and weight loss (suggestive of Johnes' disease) and have not been euthanised

  - Livestock with injuries such as cuts and broken legs that judging on the amount of swelling, secondary infection, muscle atrophy and condition loss have been ongoing for more than a day.
  - Livestock that are stuck in a fence or mud when there is a large amount of faecal material, thrashing marks on the ground or marked dehydration.

16. ***There is evidence that entire male livestock are securely managed away from female stock that are too young and /or too small to be pregnant***

Direct observations

- Young females are visibly pregnant with a frame size not suitable for pregnancy
- Males appear to be running with the mob / herd all year around
- There are offspring of all ages in the mob / herd and males present also
- Carcasses of young females present with evidence that they have died at parturition
- Young male stock are not routinely castrated and are running with the females post weaning

17. ***There is evidence that livestock in poorer condition are drafted out of the mob/herd/ flock and preferentially fed***

Direct observations

- A mob / herd of livestock in mixed condition, where some animals are unacceptably poor in condition. It is not possible to affectively preferentially feed livestock while with the mob / herd. Appropriate supplementation can only be provided when livestock can access extra nutrition in a non-competitive environment.

18. ***There is evidence that the farm is stocked with the appropriate number of livestock for the area available (not overstocked)***

Direct observations

- If there is very little if any pasture available in any of the paddocks on the farm at a time of the year when you would expect there to be some surplus, this suggest the farm is overstocked.
- When there is clearly a large number of animal's present compared to the size of the property / paddock, suggests the farm is overstocked
- A rough calculation of animals /hectare can help to quantify the stocking rate
- Visually comparing stocking rates with adjoining farms can provide a guide as to a suitable stocking rate for that area

- When the mob / herd includes a large number of animals born in the previous breeding season, this can suggest that the farmers has not sold any or many stock this might suggest the farm is overstocked
- Poor facilities for drafting and selling stock can also indicate that overstocking is likely

19. Has this property had a substantiated poor livestock welfare incident in the past? Yes / No

This includes any instance where poor livestock welfare has been substantiated and recorded on the agency data base for verification. Previous investigations that have not been substantiated should **NOT** be included here.

20. If so, how long ago?

a. Less than 1 year

b. 2-4 years

c. 5-9 years

d. 10 or more years

Have you got anything further that you would like to add?

AWRAT-D on the go summary

Access to AWRAT-D

QR code

Link

Vital reminders

- Please complete the assessment **after** you have left the farm
- Please make sure you enter a code for each visit (so I can identify revisits)
- Please complete the assessment for **every** farm visit, revisit that you conduct during the study period
- Please ensure that all participants present at the farm visit complete the survey
- **DO NOT** consult with the farmer to complete the assessment
- **DO NOT** travel around the farm beyond what is required for your visit
- **ONLY** complete the assessment for farm visits where there are nondairy: cattle, sheep or goats
- Please complete the assessment by yourself not in discussion with colleagues

If you have any questions, please contact Natarsha Williams on:

- 0422 414 964
- natarsha.williams@student.unimelb.edu.au

Thank you so much for your help to complete this project and this section of my PhD. I really appreciate your involvement.

Animal Welfare Risk Assessment Tool

Case code:

First letter of your middle name (or if not applicable, surname)

First letter of your favourite sports team (e.g. AFL, league, netball, basketball)

First 2 letters of location (town) where farm is located- ____ ____

First letter of farmers surname- ____ (if unknown put $)

Todays date ___ ___/___ ___/ ___ ___ ___ ___

Has an AWRAT-D assessment been previously completed for this property (is it a revisit)? YES/ NO

Section A: Background on survey participants

1. What state or territories in Australia do you work? (click as many as are applicable)
    - Victoria
    - New South Wales and ACT
    - South Australia
    - Western Australia
    - Tasmania
    - Queensland
    - Northern Territory

2. What is your main role?
    - Animal Welfare officer/ veterinarian
    - Veterinarian (private practice)
    - Other pls specify

Section B Farm visit details

3. What was the main purpose of your visit to this farm?
    - Disease investigation
    - Routine audit/ project work
    - Animal welfare investigation
    - Other, please specify

If animal welfare was this a revisit or repeat visit to animal welfare investigation:

If a revisit animal welfare investigation, has the welfare of the animals improved since your last visit? Yes/no

4. Was the report of poor welfare substantiated on this property during this visit?
    - Yes
    - No
5. Using the scale below mark the spot that you feel best describes the overall welfare status of the livestock on the farm during your visit today?

Very poor ______________________________ ***Very good***

Using the scale for each separate factor please indicate how relevant you think each one was on the farm.

If the statement was relevant to the farm, mark closer to the green / right and if the statement was not relevant to the farm mark closer to the red / left. If you are unsure of you were unable to verify any of the factors please mark the *'I don't know / I am unable to verify this'*

Not true*___________________________|______ *Very true

I don't know / I am unable to verify this ☐

Farm

1. There is evidence that the farm fencing is of an effective standard (e.g. stock proof, swinging gates)
2. There is evidence that the stock handling facilities are suitable and in working order
3. There is evidence that the feed and water areas and facilities are clean, functional and accessible to all livestock
4. There is evidence that the farm has an equivalent amount of pasture (or better) to that on surrounding farms
5. There is evidence that paddocks with livestock are suitable to avoid illness and accidental injury (e.g. toxic plants, feeding off the ground)
6. There is evidence that dead animals are removed from paddocks

Nutrition

7. There is evidence that livestock are fed according to their reproductive state (e.g. empty, pregnant or lactating)
8. There is evidence that some paddocks have good feed remaining
9. There is evidence that the feed offered is suitable for the class of animal (e.g. energy, protein etc)
10. There is evidence that supplementary feeding is offered before significant weight loss occurs
11. There is evidence that there is some supplementary feed remaining in the paddock after the livestock have finished feeding
12. There is evidence that all animals have equal access to supplementary feeding (e.g. well spread out or continuous access)

Husbandry and management

13. There is evidence that the lambs, calves and / or kids within a mob / herd are all a similar age (within 2-3 months of each other)
14. There is evidence that animals with conditions that are unresponsive or not economical to treat are humanely culled without delay (e.g. lameness, cancers, chronic scouring, congenital abnormalities)

15. There is evidence that the investigation and treatment of health conditions affecting livestock are completed as soon as the problem is identified
16. There is evidence that entire male livestock are securely managed away from female stock that are too young and /or too small to be pregnant
17. There is evidence that livestock in poorer condition are drafted out of the mob/herd/ flock and preferentially fed
18. There is evidence that the farm is stocked with the appropriate number of livestock for the area available (not overstocked)

Historical investigations

19. Has this property had a substantiated poor livestock welfare incident in the past? Yes / No
20. If so, how long ago?
    - Less than 1 year
    - 2-4 years
    - 5-9 years
    - 10 or more years

Have you got anything further that you would like to add?

Observation study

Intra and inter-observer test

The purpose of this study is to compare how observers rate a series of pictures of various features / situations on a farm using a sliding scale, **on two occasions 10-14 days apart**. This will assess the consistency of observer assessment given the same images in the same testing environment but at different times (10-14 days apart).

Eligibility criteria: Participants must be at least 18 years old and have worked with non-dairy: cattle, sheep and goats for at least 6 months.

Please complete both tests on a computer or tablet rather than a phone to ensure the images are large enough to view adequately to make an assessment.

Please make an assessment based on the information in the picture alone, without extrapolating as to what the rest of the farm may be like. Some of the pictures will show features that are very obviously of a great standard and others not so much. This test aims to compare how similarly an observer rates the same set of images / situations when repeated 10-14 days apart. There is no wrong or right answer. It may be difficult to see a lot of detail in the pictures, please make your best judgement based on what you can observe.

This test does not collect identifying information but does ask you to provide a code so paired tests can be compared.

1. Participant Code
    - Please enter the first letter of your middle name (or if not applicable, surname)
    - Please enter the first letter of your favourite sports team (e.g. AFL, league, netball, basketball)
    - Please enter your year of birth

E.g. WH27061969

2. What is todays date?

3. What state or territories in Australia do you work? (click as many as are applicable)
    - Victoria
    - New South Wales and /or ACT
    - South Australia
    - Western Australia
    - Tasmania
    - Queensland
    - Northern Territory

4. What is your main role?

- Animal Welfare officer/ veterinarian
- Veterinarian (private practice)
- Livestock extension officer
- Stock agent
- Producer
- Other please specify

Intra and inter-observer test

For the following questions please consider the photo/s provided and mark a rating on the scale that best applies to the question posed. If the statement is accurate or true in respect to what you can see in the picture mark closer to the green / right and if the statement is not accurate or wrong, mark closer to the red / left.

Not accurate ___________________________ ***Very accurate***

Farm

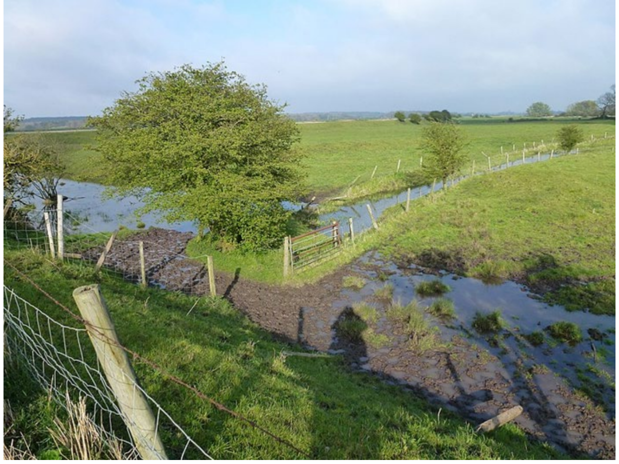

Photograph by Richard Humphrey under the Creative Commons licence

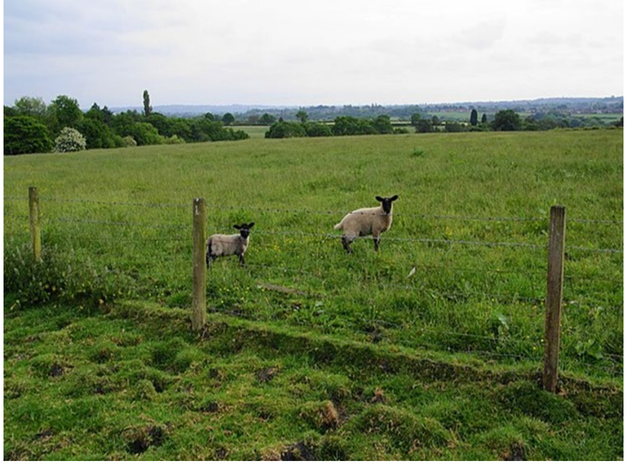

Photograph by Bill Boaden under the Creative Commons licence

1. **For a sheep enterprise there is evidence that the farm fencing is of an effective standard (e.g. stock proof, swinging gates)**

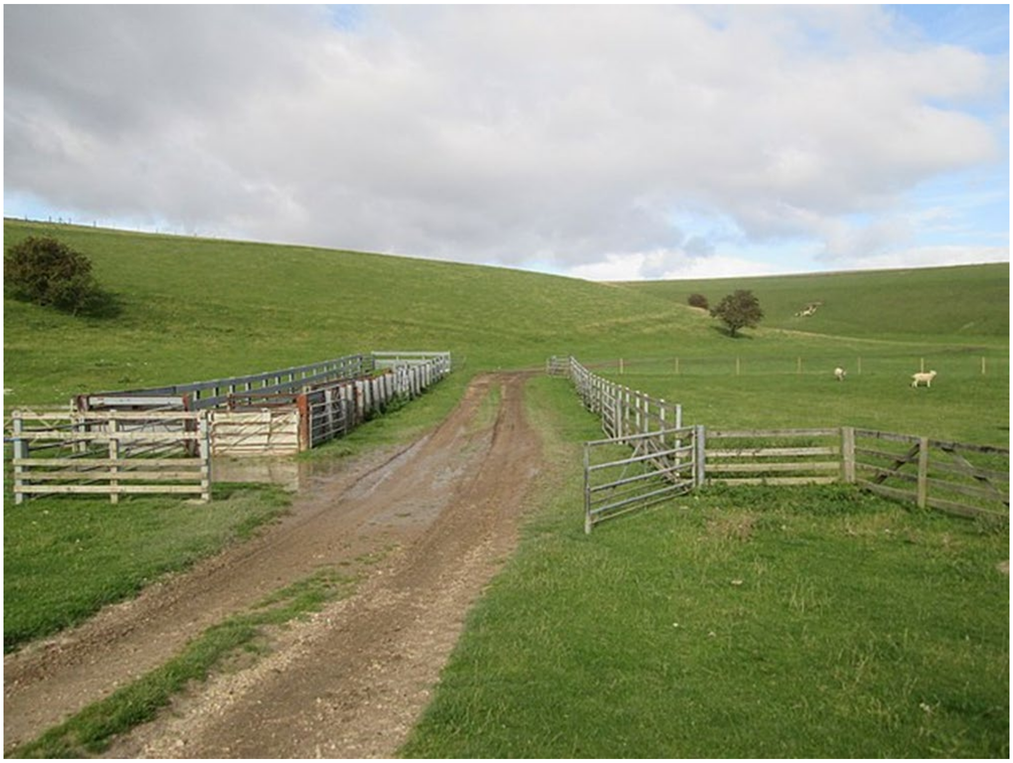

Photograph by [Martin Dawes](#) under the [Creative Commons licence](#)

2. For a sheep enterprise there is evidence that the farm fencing is of an effective standard (e.g. stock proof, swinging gates)

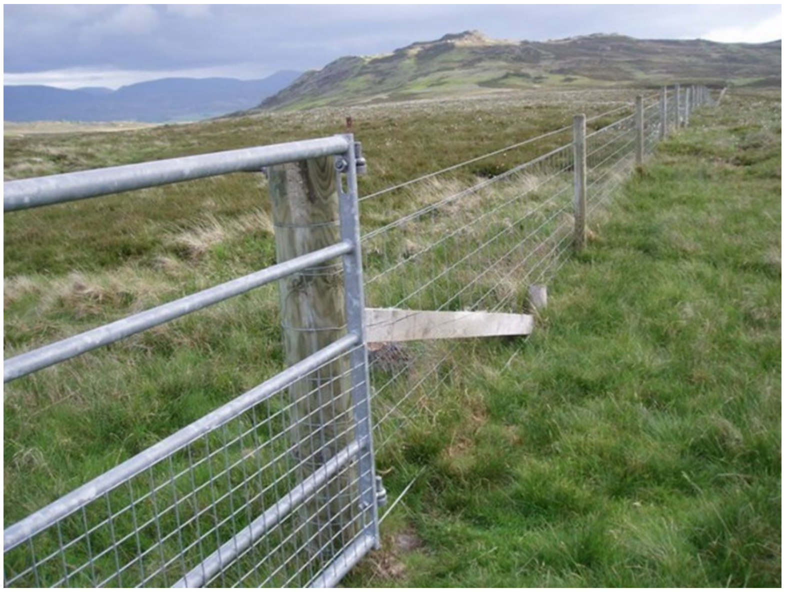

Photograph by Dorothy Carse under the Creative Commons licence

3. For a sheep enterprise, there is evidence that the farm fencing is of an effective standard (e.g. stock proof, swinging gates)

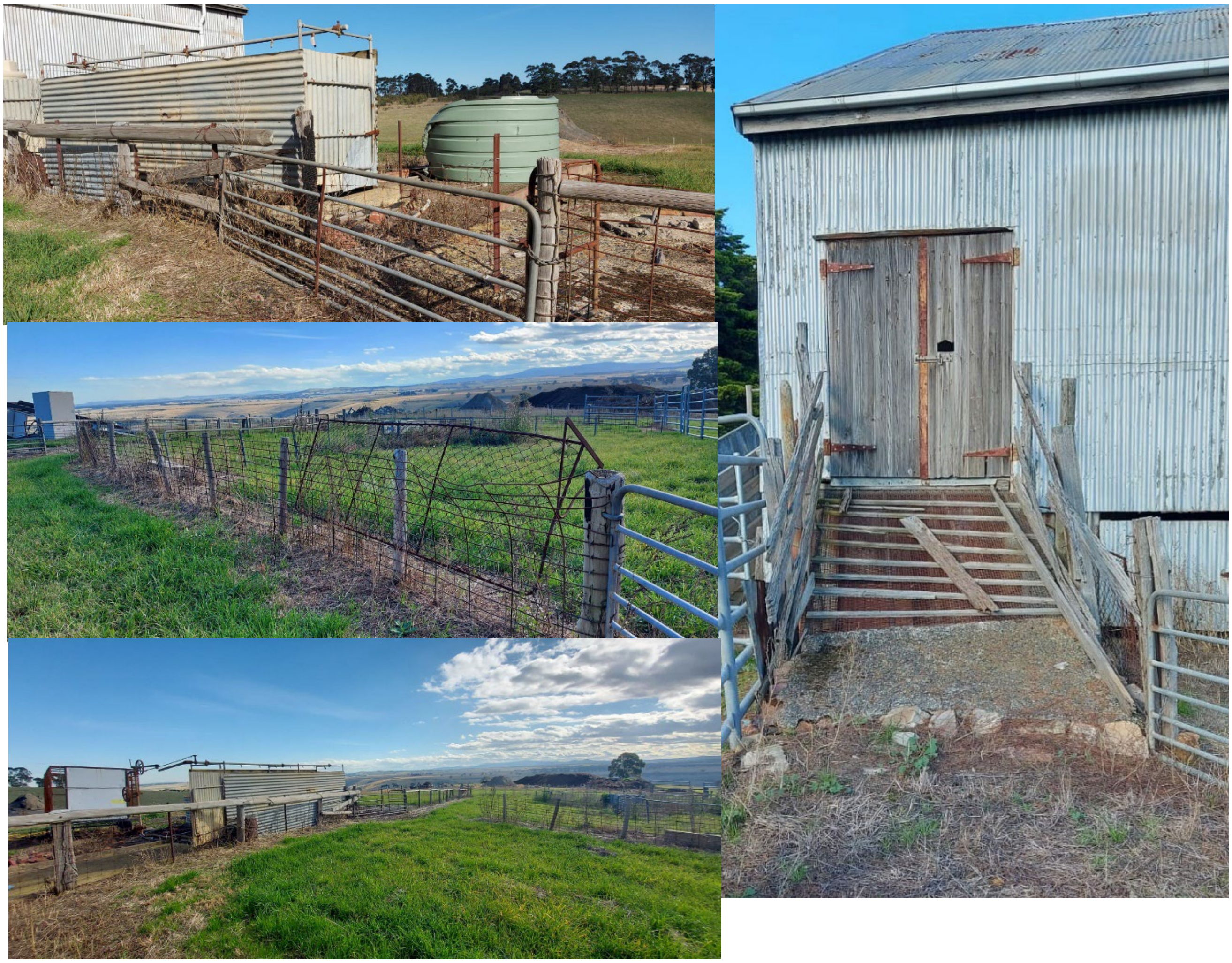

Photographs with permission from anonymous source

4. For a sheep enterprise there is evidence that the stock handling facilities are suitable and in working order

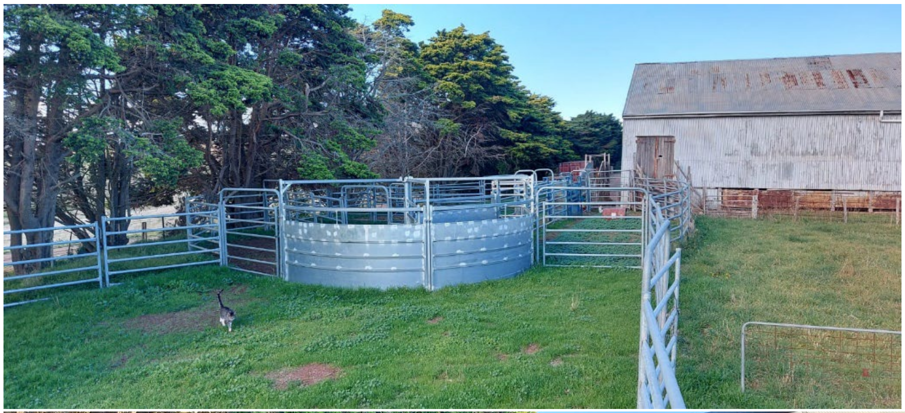

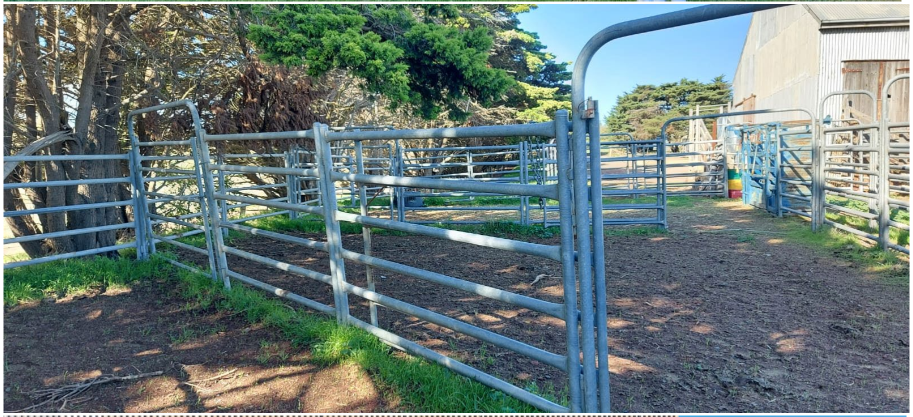

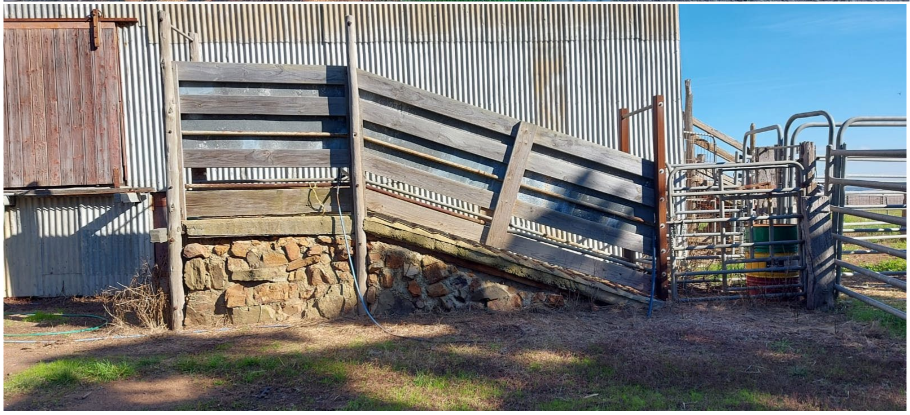

Photographs with permission from anonymous source

5. For a cattle enterprise there is evidence that the stock handling facilities are suitable and in working order

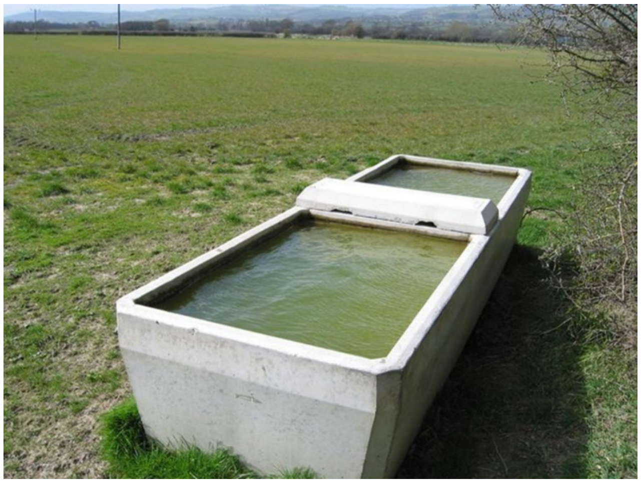

Photograph by [Dave Croker](#) under the [Creative Commons licence](#)

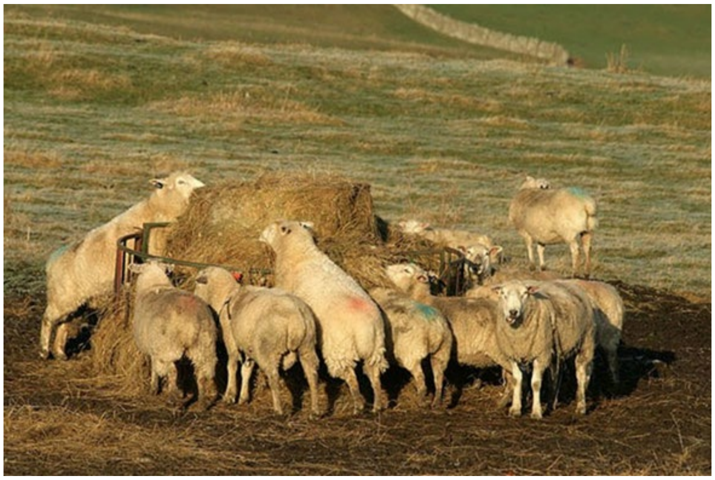

Photograph by [Walter Baxter](#) under the [Creative Commons licence](#)

6. There is evidence that the feed and water areas and facilities are clean, functional and accessible to all livestock

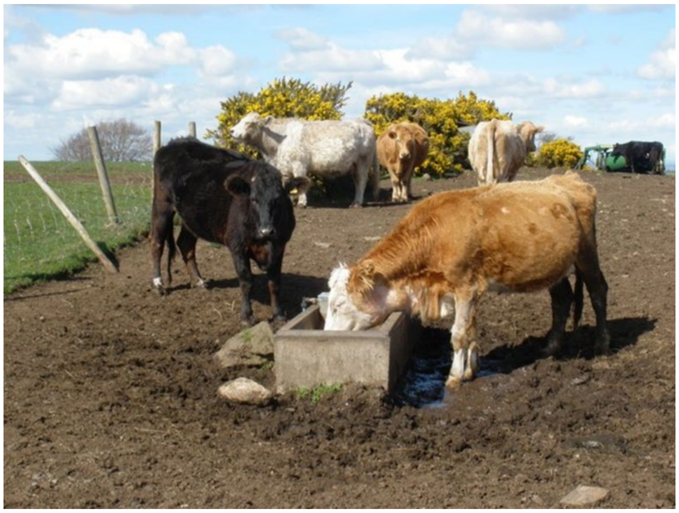

Photograph by [Roger Cornfoot](#) under the [Creative Commons licence](#)

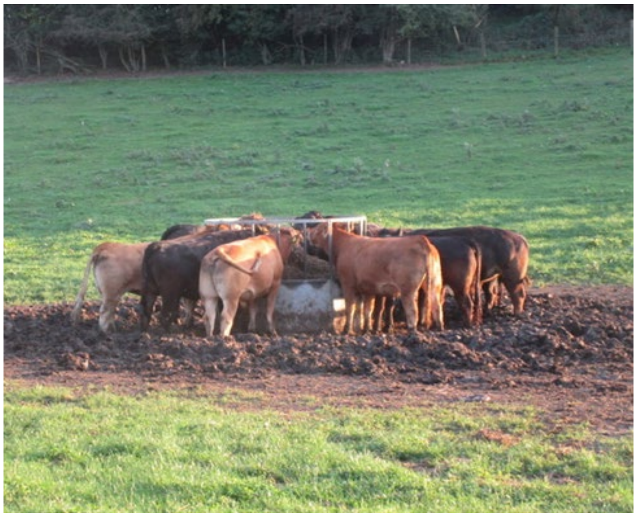

Photograph by [John Thacker](#) under the [Creative Commons licence](#)

7. There is evidence that the feed and water areas and facilities are clean, functional and accessible to all livestock

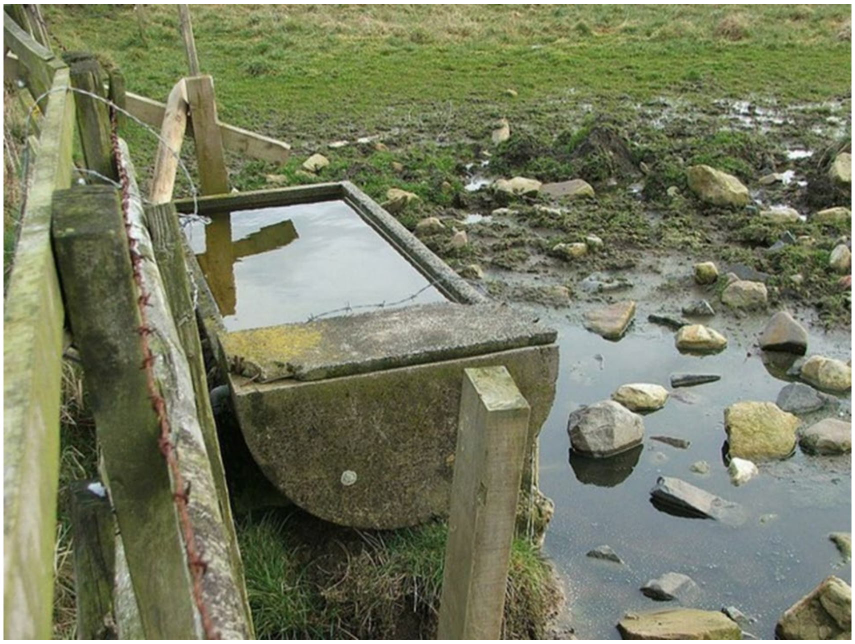

Photograph by [Christine Westerback](#) under the [Creative Commons licence](#)

8. **There is evidence that the feed and water areas and facilities are clean, functional and accessible to all livestock**

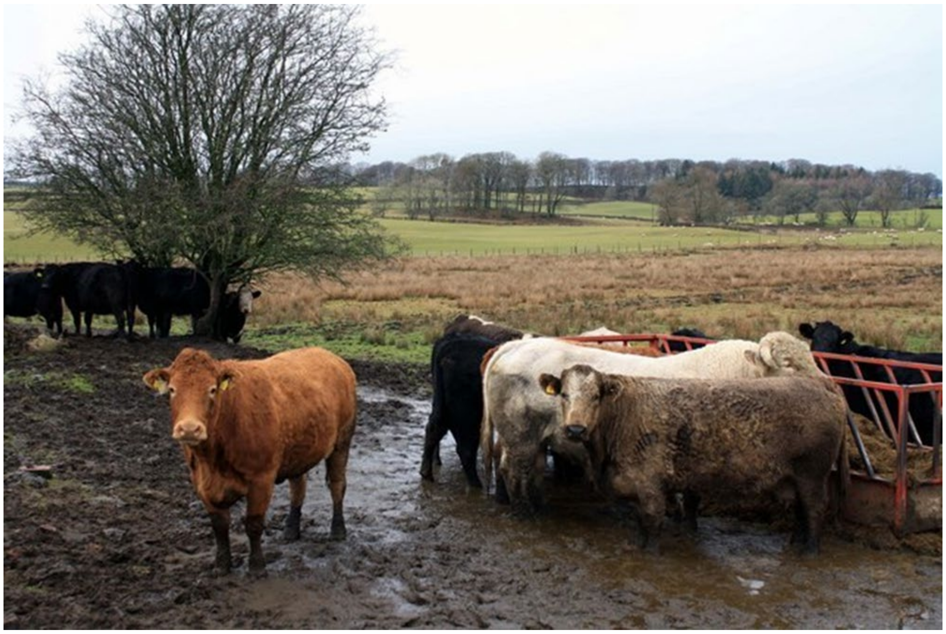

Photograph by [Colin Kinnear](#) under the [Creative Commons licence](#)

9. **There is evidence that the feed and water areas and facilities are clean, functional and accessible to all livestock**

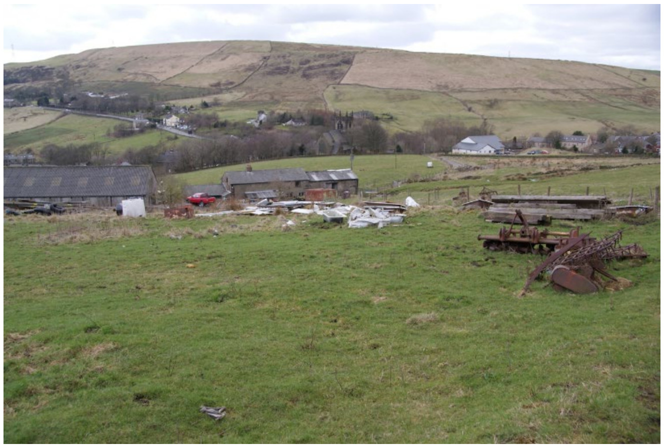

Photograph by Bill Boaden under the Creative Commons licence

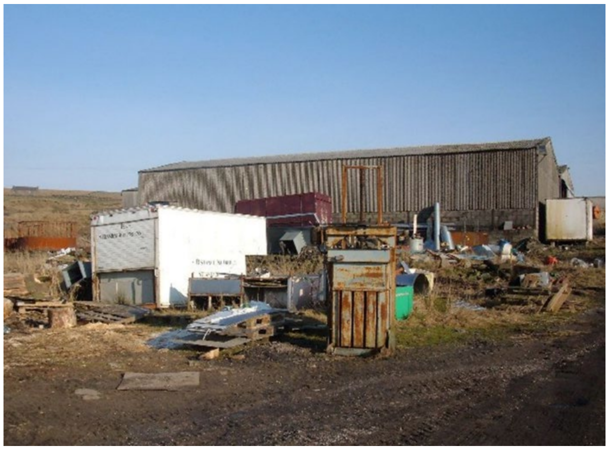

Photograph by Michael Ely under the Creative Commons licence

10. There is evidence that the paddocks with livestock are suitable to avoid illness (e.g. toxic plants, feeding off the ground)

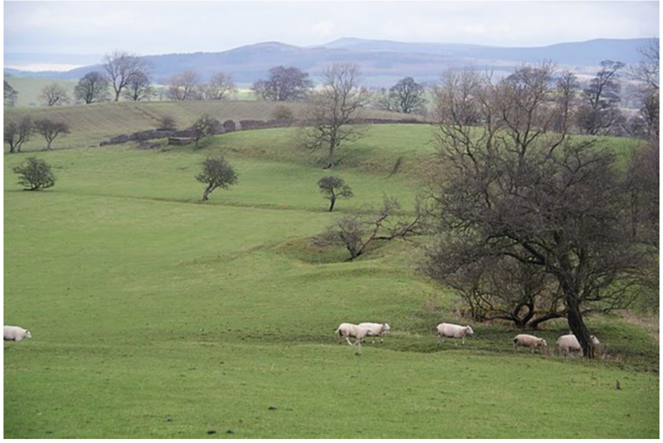

Photograph by *[Bill Boaden](#)* *under the* *[Creative Commons licence](#)*

11. There is evidence that the paddocks with livestock are suitable to avoid illness (e.g. toxic plants, feeding off the ground)

Nutrition

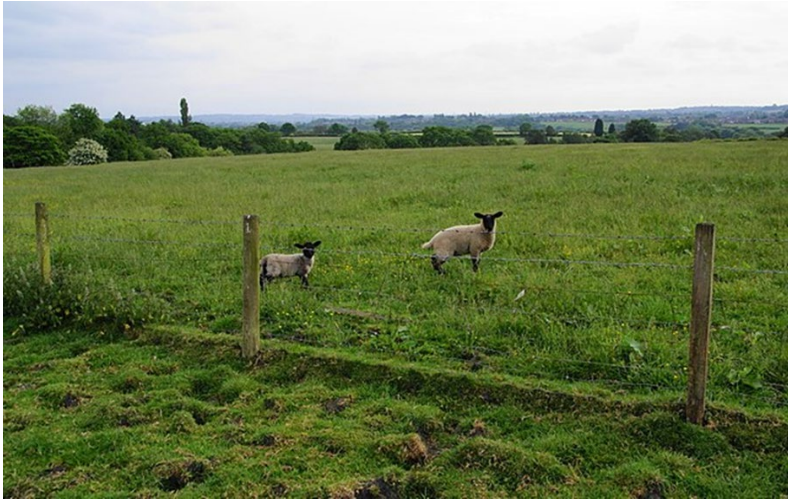

Photograph by *Bill Boaden* *under the* *Creative Commons license*

12. There is evidence that some paddocks have good feed remaining

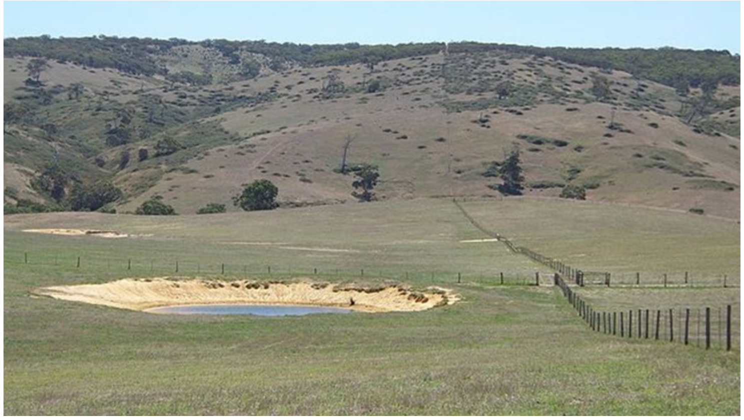

Photograph by [Doug Butler] under the [Creative Commons licence]

Assuming all of the farm is captured in this picture

13. There is evidence that some paddocks have good feed remaining

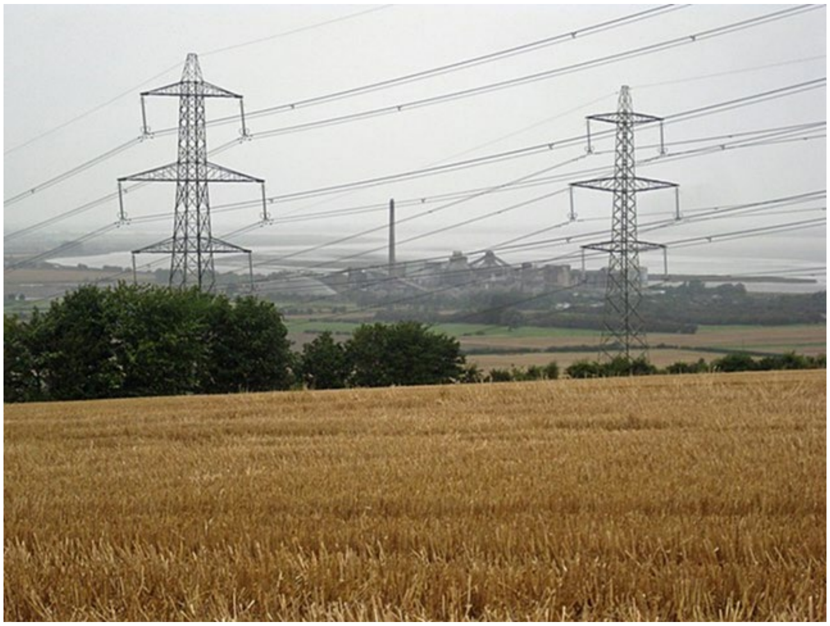

Photograph by [David Wright] under the [Creative Commons licence]

Assuming calving cows are grazing this paddock

14. There is evidence that the feed offered is suitable for the class of animal (e.g. energy, protein etc)

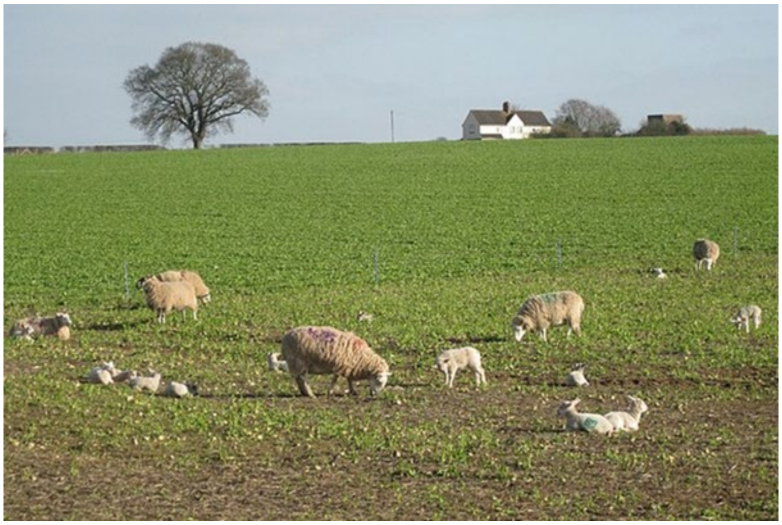

Photograph by Robin Stott under the Creative Commons licence

15. There is evidence that the feed offered is suitable for the class of animal (e.g. energy, protein etc)

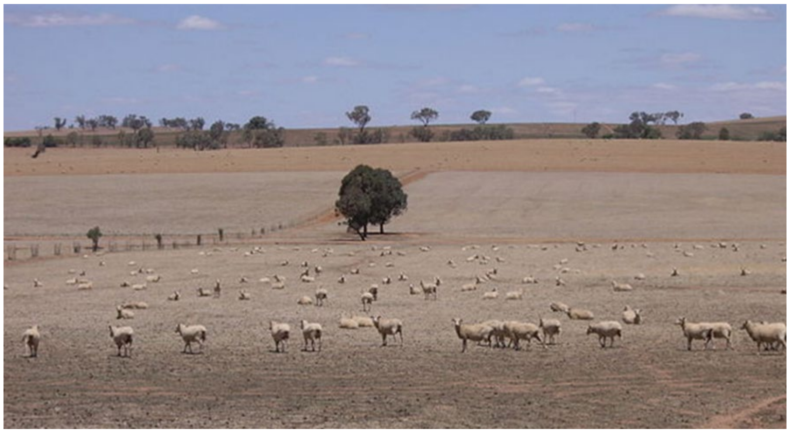

Photograph by VirtualSteve under the Creative Commons licence

16. There is evidence that supplementary feeding is offered before significant weight loss occurs

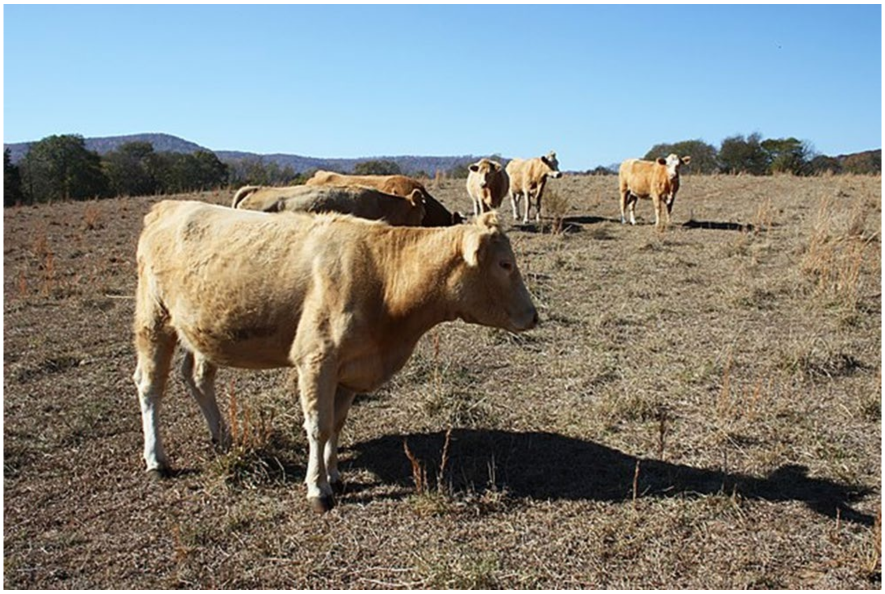

Photograph by [Alabama Extension] under the [Creative Commons licence]

17. There is evidence that supplementary feeding is offered before significant weight loss occurs

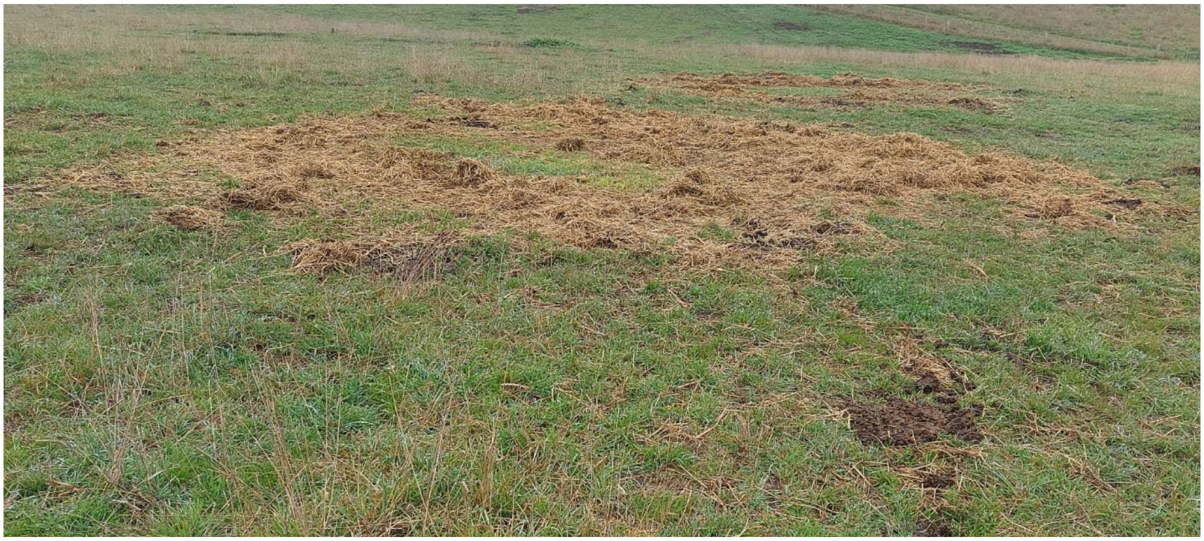

Photographs accessed with permission from an anonymous source

18. There is evidence that some supplementary feed remains in the paddock after the livestock have finished feeding

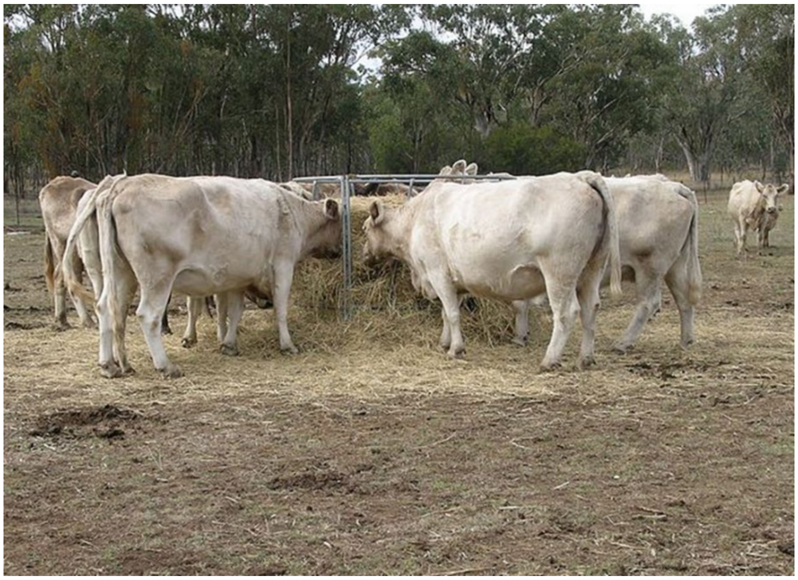

Photograph by [C.Goodwin]() under the [Creative Commons licence]()

19. There is evidence that all animals have equal access to supplementary feeding (e.g. well spread out or continuous access)

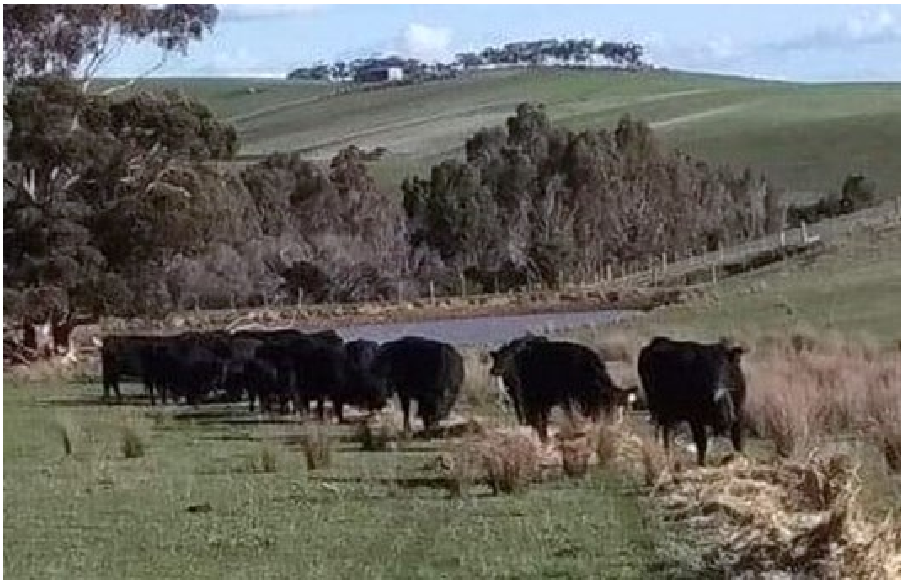

Photograph provided with permission from anonymous source

20. There is evidence that all animals have equal access to supplementary feeding (e.g. well spread out or continuous access)

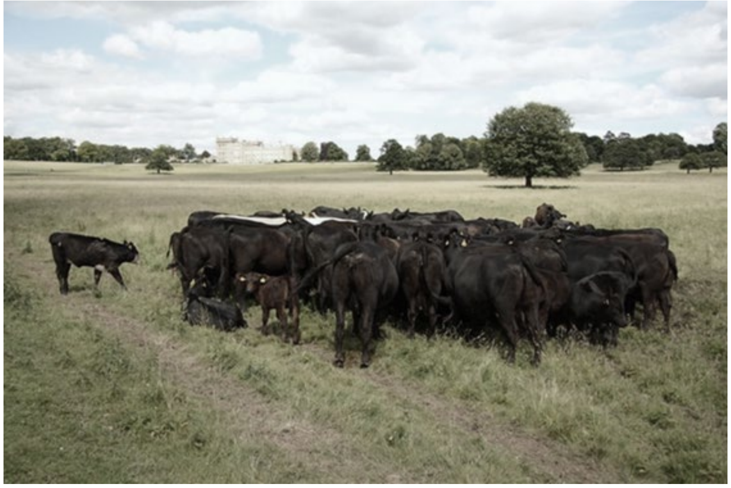

Photograph by [Richard Croft](#) under the [Creative Commons licence](#)

21. There is evidence that all animals have equal access to supplementary feeding (e.g. well spread out or continuous access)

Management and Husbandry

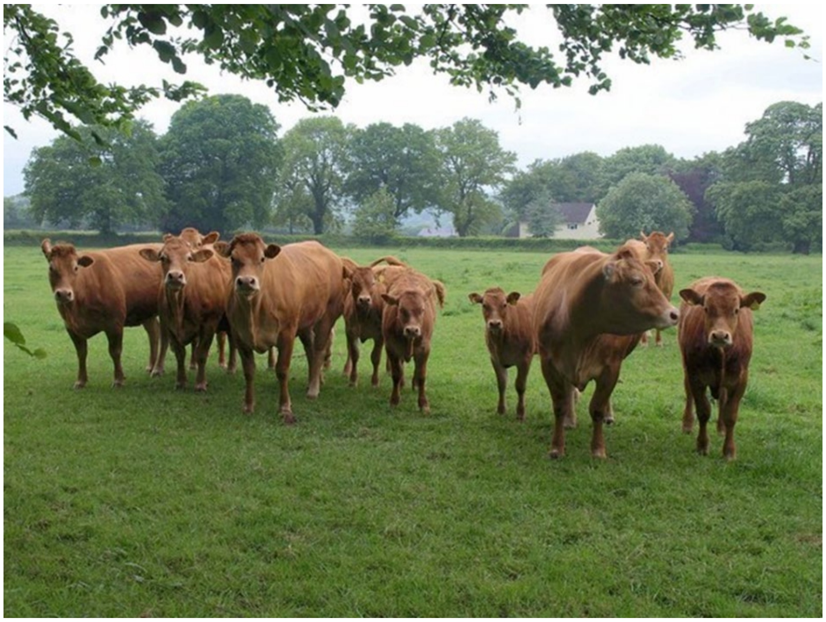

Photograph by [Derek Harper](#) under the [Creative Commons licence](#)

22. There is evidence that the lambs, calves and / or kids within a mob / herd are all a similar age (within 2-3 months of each other)

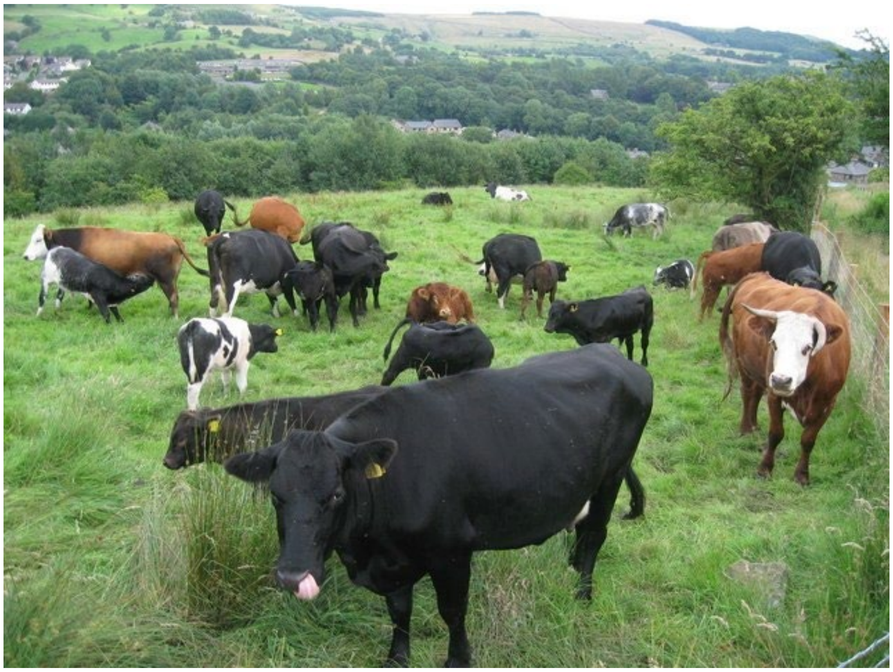

Photograph by [Paul Anderson](#) under the [Creative Commons licence](#)

23. There is evidence that the lambs, calves and / or kids within a mob / herd are all a similar age (within 2-3 months of each other)

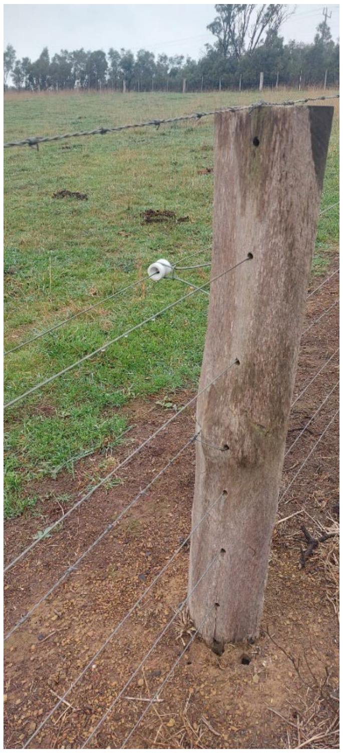

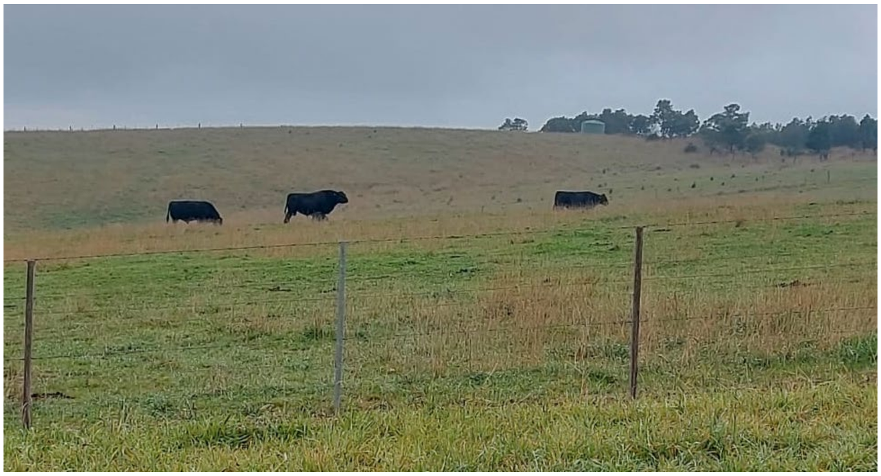

Photograph accessed with permission from an anonymous source

24. There is evidence that the entire male livestock are securely managed away from female stock that are too young and /or too small to be pregnant
